# Case Study on Formulating a Multidose Lyophilized Measles-Rubella (MR) Vaccine Candidate for Local Manufacturing in Africa: Lab-Scale Results and Lessons Learned for Future Technology Transfer

**DOI:** 10.3390/vaccines14100900

**Published:** 2026-10-14

**Authors:** Kathryn Secrist, Prashant Kumar, Michael Z. Wang, Soraia Saleh-Birdjandi, Sagar Salave, Ozan S. Kumru, Ngana Salem Mberkadji, Fama Sakho, Sangeeta B. Joshi, David B. Volkin

**Affiliations:** 1 Department of Pharmaceutical Chemistry, Vaccine Analytics and Formulation Center, The University of Kansas, Lawrence, KS 66047, USA; 2 Vaccine Research Center (VRC), Institut Pasteur de Dakar (IPD), Dakar B.P. 220, Senegal

**Keywords:** Measles, Rubella, vaccine, formulation, stability, lyophilization, qPCR, viral infectivity assay, excipients

## Abstract

**Background/Objectives:** Local manufacturing of Measles-Rubella (MR) vaccines is part of a multifaceted approach to improve vaccine accessibility in Africa, yet process development and technology transfer challenges remain for large-scale production of viral bulks and lyophilized drug product. This case study describes lab-scale formulation development of a multidose, lyophilized MR vaccine candidate, leveraging the availability of concentrated bulks of Measles (MeV) and Rubella (RuV) produced in MRC-5 cells by a new bulk manufacturing process. **Methods:** Formulation studies utilized both high-throughput RT-qPCR viral infectivity assays and standard lyophilization measurements. The off-patent, publicly available formulation composition of a single-dose, lyophilized trivalent MMR vaccine was evaluated as a starting point to develop a multidose, bivalent lyophilized MR vaccine candidate. To identify optimized conditions, various formulation (e.g., human serum albumin, viral bulk volume), drug product (e.g., vial size, fill volume), and lyophilization (e.g., freezing rate, primary/secondary drying cycle) parameters were evaluated to lower costs and assess MR stability during lyophilization, storage, and reconstitution. **Results:** Real-time and accelerated stability studies were conducted with four variations of the top candidate MR formulation, filled into primary packaging selected for technology transfer and lyophilized in both lab- and pilot- scale freeze-dryers. An optimized five-dose MR vaccine formulation with 5% (*v*/*v*) bulk and 95% (*v*/*v*) stabilizer solution, filled at 2X and reconstituted to 1X concentrations, demonstrated promising stability profiles and cost reduction potential. Stability profiles at 2–8 °C were used to estimate the MR release doses needed to ensure the required expiry doses at end of shelf-life. **Conclusions:** Results are discussed, along with recommendations for future work, in the context of the analytical, formulation, and lyophilization challenges encountered during lab-scale development of a candidate multidose, lyophilized MR vaccine dosage form targeted for technology transfer and large-scale local production in Africa.

## 1. Introduction

Measles (MeV; *Paramyxoviridae*) and Rubella (RuV; *Matonaviridae*) viruses are both highly contagious pathogens transmitted by aerosol and respiratory droplets [1,2]. MeV remains a leading cause of childhood mortality worldwide [3], while RuV poses serious risks to pregnant women, including miscarriage, stillbirth, and congenital Rubella syndrome (CRS) [4]. Live-attenuated vaccines for Measles and Rubella (MR) have been widely used for decades to combat these diseases. Currently available vaccines are highly effective; for example, the M-M-R^®^II vaccine has demonstrated over 90% efficacy with a two-dose vaccine regimen and is estimated to have prevented over 20 million deaths since 2000 [5]. Examples of MR-containing vaccines include monovalent preparations (MeV or RuV) as well as different combinations including bivalent (MR), trivalent (MR with mumps, MMR) and quadrivalent (M, R, mumps, varicella, MMRV) [6,7].

The WHO African Region (AFRO) strives to improve Measles and Rubella (MR) vaccine coverage to achieve MR elimination in 80% of countries by 2030. Despite dedicated global efforts to expand MR vaccine coverage, achieving the key targets of the WHO global Immunization Agenda 2030 (IA2030) [8,9] remains a challenge. Since primary vaccine failures have occurred in a proportion of individuals following the first dose of Measles-containing vaccine, a second dose is given to provide an additional opportunity for seroconversion and increased population-level immunity, which is essential for improved vaccine coverage in the African Region [10,11]. Briefly, in 2024, 84% of children globally received 1 dose, and only 76% received a second dose of Measles-containing vaccine, while only 73% received one dose of Rubella-containing vaccine in their national immunization schedules [8]. Amongst all six WHO regions, the African region (AFRO) still bears a large burden of vaccine-preventable MR diseases and has the lowest coverage rates compared to other regions [12]. A recent study reported that in 2023, 74% of vaccinated children aged 1–2 in the African region possessed vaccine-induced protection against Measles. Such protection levels are insufficient to prevent wild-type MeV transmission given the virus’s basic reproduction number (R_0_) ≥ 10 [13]. One approach to facilitate global eradication, particularly in low- and middle- income countries (LMICs), is to enable local production of MR-containing vaccines; however, there are many hurdles, including limited local infrastructure, financing, and expertise, as well as technical challenges related to technology transfer and large-scale manufacturing of MeV and RuV bulks and the lyophilized MR drug product [5,14].

The multidose, lyophilized MR vaccine formulation development work described in this case study was part of a larger, collaborative effort to enable technology transfer and local production of a live-attenuated, MR vaccine candidate in Africa [15,16,17,18,19,20]. The consortium, funded by grants from the Gates Foundation, consisted of key partners, namely, (1) Institut Pasteur de Dakar (IPD) in Senegal as the project lead and the site for future commercial production of MeV and RuV bulks and the MR lyophilized vaccine, and (2) Batavia Biosciences (BBS) in the Netherlands that produced concentrated high titer MeV and RuV viral bulks in MRC-5 cells using a new bulk manufacturing process, and was responsible for technology transfer of a GMP manufacturing process to produce MeV and RuV viral bulks at large-scale at IPD [15,17,18,21,22]. The role of the Vaccine Analytics and Formulation Center (VAFC) at the University of Kansas (KU), the third collaborator, was to identify and address (at lab-scale) potential formulation, lyophilization, and stability challenges to “de-risk” future drug product technology transfer and production at IPD of a lyophilized, multidose MR vaccine candidate.

The formulation development of live, attenuated viral vaccines (LVVs) in general, and MR-containing vaccines in particular, focuses on overcoming their inherent lability. The instability of enveloped viruses in LVVs is well-documented and primarily attributed to water-mediated degradation pathways, including structural alterations of the lipid envelop structural and surface proteins, and/or nucleic acid components [23,24,25]. MR-containing vaccines typically require stringent cold chain maintenance [24] for the viral bulks (e.g., as a frozen liquid aliquoted in large volume containers stored at ultralow temperatures, i.e., <−60 °C) and the final vaccine drug product (e.g., filled into individual glass vials, lyophilized, and stored at 2–8 °C) [26]. Lyophilization enhances viral stability during long-term storage in the refrigerator by (1) removing bulk water that mediates viral degradation, and (2) avoiding exposure to elevated temperatures during drying (i.e., drying by sublimation of ice under controlled temperatures and pressures). Lyophilization results in preserving viral potency by immobilizing viral particles within a stabilizing matrix and replacing water-mediated hydrogen bonds with those of excipients to retain structural integrity [23,24,27]. Many commercial preparations of LVVs, including all MR-containing vaccines, are manufactured as a lyophilized dosage form, requiring addition of a stabilizer solution prior to freeze-drying as well as a separate diluent for reconstitution upon administration [23,27,28].

One key LVV formulation challenge is that the removal of bulk water during freeze-drying, and subsequent storage in the solid state, causes loss of potency in the absence of stabilizing excipients [29]. Typically, complex mixtures of additives are required to provide sufficient viral stabilization [23]. For example, the commercial lyophilized M-M-R^®^II formulation contains many excipients [30], with some components difficult to lyophilize; e.g., culture media and sorbitol both have very low glass transition temperatures (*T_g_*′) [31]. Freeze-drying must therefore be carried out at very cold temperatures (e.g., −35 °C or below), resulting in slow sublimation rates and long lyophilization cycles (e.g., multiple days). In summary, developing a stabilizing formulation linked to an effective lyophilization cycle is a key part of LVV formulation development requiring careful optimization of (1) the solution properties (e.g., glass transition temperature [*T_g_*′], osmolality, viscosity), (2) freeze-drying parameters (e.g., primary and secondary drying cycles, equipment settings, total cycle times), (3) pharmaceutical properties after freeze-drying (e.g., residual moisture, reconstitution time, cake appearance), and (4) viral infectivity titers, including lyophilization yields and stability profiles during storage at various temperatures [31,32].

The formulation development of a lyophilized, multidose MR vaccine candidate targeted for production and use in the AFRO region first required consensus within the consortium to define formulation aspects of the Target Product Profile (TPP) [33,34,35], including the multidose requirements (number of doses), primary packaging (e.g., vials, stoppers, diluent), and storage goals (e.g., expiry dose, storage temperature and shelf-life, VVM designation). Based on these TPP requirements, several key goals for this case study on multidose MR vaccine formulation development were established including (1) developing and implementing analytical assays for MR formulation development, (2) defining the composition of the MR lyophilized multidose vaccine candidate based on an off-patent, commercially-used formulation with publicly-available composition information, (3) developing a suitable fill volume and lyophilization cycle for freeze-drying of the multidose formulation, and (4) performing real-time and accelerated stability studies to establish the “stability window” of an MR vaccine candidate (i.e., establish the vaccine release dose to ensure the required vaccine expiry dose after lyophilization, storage, and reconstitution stability). Additional formulation development work, performed outside the scope of these objectives and as described in a companion paper [36], focused on systematic excipient screening, formulation optimization, and stability studies to identify a new MR vaccine formulation (using lower-cost, non-animal-derived excipients). Finally, key experimental results from this case study are discussed, and recommendations for future work are provided, in the context of future translation of this lab-scale work to large-scale, local production in Africa.

## 2. Materials and Methods

### 2.1. Materials

Concentrated viral bulks (one lot for each virus) of live, attenuated Measles (MeV; Edmonston–Zagreb strain at 6.6 log TCID_50_/mL) and Rubella (RuV; Wistar RA 27/3 strain at 7.1 log TCID_50_/mL) were obtained from Batavia Biosciences (BBS, Leiden, The Netherlands). The MeV and RuV vaccine strains were grown in MRC-5 cells via a new manufacturing process in bioreactors followed by downstream processing steps including harvesting, clarification, concentration, and filtration as described elsewhere [16,17]. The RuV bulk contained approximately 1.8 mg/mL of recombinant human serum albumin (rHSA), whereas the MeV bulk was produced without rHSA. The estimated levels of excipients and manufacturing process residuals in the final MeV and RuV bulks were provided by Batavia Biosciences (BBS, Leiden, The Netherlands), and this information was subsequently used to estimate the composition of the candidate multidose Measles-Rubella (MR) formulation described in this study.

Sucrose (catalog#: S-124-1-MC) was sourced from Pfanstiehl (Waukegan, IL, USA), and rHSA (Exbumin, catalog# 777HSA097) was purchased from InVitria, Inc. (Junction City, KS, USA). Other key analytical reagents and formulation excipients including D-sorbitol (catalog# S7547), hydrolyzed gelatin (H. gel, catalog# G0262), L-glutamic acid monosodium salt (MSG, catalog# 49621), potassium phosphate (monobasic, catalog# P0662; and dibasic, catalog# P3786), sodium bicarbonate, and sodium phosphate (monobasic, catalog# S9638; and dibasic, catalog# S9763) were obtained from Sigma-Aldrich (St. Louis, MO, USA). For the viral infectivity PqPCR assay, Vero cells (catalog #: CCL-81) were obtained from ATCC (Manassas, VA, USA), TaqPath™ 1-Step Multiplex Master Mix (catalog# A28527) was obtained from ThermoFisher Scientific (Waltham, MA, USA), and all primers and probes were obtained from IDT Technologies (Coralville, IA, USA) except the RuV probe, which was obtained from Thermo Fisher Scientific (Waltham, MA, USA).

The lyophilized, multidose, bivalent MR formulation composition (referred to as the candidate MR formulation) described in this work was developed using, as a starting point, the publicly-available formulation composition of the off-patent, commercially available, single dose, trivalent M-M-R^®^II vaccine, as described in the vaccine’s product monograph [30]. Briefly, the candidate multidose MR formulation in this work differed from the single-dose M-M-R^®^II formulation as follows: (1) different MeV virus attenuated strains, and both MeV and RuV were grown in different host cell substrates using different bulk processes, (2) certain excipients were removed or minimized to lower costs and facilitate excipient procurement (see Section 3), (3) the concentrated MeV and RuV viral bulks differed in composition (e.g., cell culture media composition and manufacturing process residuals) compared to those reported for the M-M-R^®^II viral bulks [30,37,38], (4) contained 5% (*v*/*v*) of viral bulks/culture media, as it used concentrated viral bulks, compared to 25% (*v*/*v*) viral bulks/culture media reported for in M-M-R^®^II [30,37,38], (5) the Mumps virus (and associated components from the viral bulks) was excluded as this work focused on a bivalent vaccine candidate, and (6) the multidose formulation was filled and lyophilized using larger glass vials and fill volumes compared to a single-dose formulation. Sterile glass vials (vial sizes 2R, 4R, 6R) were obtained from Schott (Duryea, PA, USA) or provided by IPD (Dakar, Senegal), and were stoppered with rubber stoppers and crimped with aluminum seals (provided by West (Exton, PA, USA) or IPD (Dakar, Senegal)). Graphs were plotted using Origin, Version 2020, OriginLab Corporation (Northampton, MA, USA).

### 2.2. Methods

#### 2.2.1. Thermal Glass Transition Measurements

Modulated differential scanning calorimetry (mDSC) was performed using a DSC Q100 (TA Instruments, New Castle, DE, USA) to measure the glass transition temperature values of the maximally freeze-concentrated formulated liquid MR bulks (*T_g_*′) and the lyophilized drug product (*T_g_*). Samples were hermetically sealed in aluminum pans (DSC consumables) and subjected to thermal profiles consisting of a linear temperature ramp superimposed with sinusoidal modulation. For *T_g_*′ measurements of the formulated liquid samples, the temperature was equilibrated at −70 °C, modulated ±1 °C every 60 s, held isothermal for 5 min, and then ramped to 15 °C at 3 °C/min. For *T_g_* determination of the lyophilized samples, the temperature was equilibrated at 5 °C, modulated ±1.5 °C every 60 s, held isothermal for 5 min, followed by a ramp to 200 °C, then cooled to 5 °C, and reheated to 200 °C at a rate of 3 °C/min. Reverse heat flow data were collected and analyzed to determine the *T_g_*′ and *T_g_*, defined as the midpoint of the change in heat flow (reversible component for *T_g_*) during the transition.

#### 2.2.2. Pharmaceutical Cake Quality Assessment

Lyophilized vials of various candidate MR formulations were examined visually using an Apollo II Liquid Viewer (Haywards Heath, Adelphi, UK) against a black background and photographed. Cake morphology was assessed using a three-point scale: 3 = excellent, 2 = good-to-fair, and 1 = poor.

#### 2.2.3. Reconstitution Time of Lyophilized Candidate MR Formulations

Lyophilized samples were reconstituted in pre-calculated amounts of water for injection (WFI) (Cytiva, Marlborough, MA, USA). Briefly, 0.65 mL of water was added for single-dose (lyophilized in 2R vials) and 3 mL for five-dose (lyophilized in 4R or 6R vials) presentations without any purposeful agitation, and time was recorded for the solution to appear as follows: (1) lack of visually observable solids, and (2) the general cloudiness had cleared.

#### 2.2.4. Residual Moisture Content Analysis of Lyophilized Candidate MR Formulations

Residual moisture content of lyophilized samples was measured using Karl Fischer titration with a Metrohm 756 KF coulometer with a compact oven (Metrohm, Riverview, FL, USA). The instrument was conditioned using three blank vials and two water standards before sample analysis. Approximately 50 mg of lyophilized sample was placed in glass vials and heated to 150 °C. Dry nitrogen gas (75 mL/min) carried the released water vapor into the titration vessel, where it reacted with electrochemically generated iodine. The residual moisture content was calculated from the electricity needed to produce iodine, which is stoichiometrically proportional to the amount of water present in the reaction.

#### 2.2.5. Preparation of Various Versions of the Multidose MR Formulation

A concentrated stabilizer solution (1.05X for preparing 1X formulation, 2.11X for preparing 2X formulation) was prepared by dissolving calculated amounts of excipients in WFI (Cytiva, Marlborough, MA, USA; see details in Section 3). After dissolution, the pH was adjusted to 6.2–6.6, followed by vacuum filtration using a 0.22 µm SteriCup filter (Millipore Sigma, Lenexa, KS, USA) under a biosafety cabinet, and stored at 2–8 °C until further use. Calculated volumes of MeV and RuV bulks (based on target titers for a particular experiment) and MEM were then added in a biosafety cabinet and gently mixed to prepare the following final composition of the MR formulation: (1) 1X formulation excipient composition (after reconstitution), (2) target viral titers of MeV and RuV (after reconstitution), (3) 5% (*v*/*v*) of viral bulks (including any additional culture media used to dilute the viral bulks to the target concentration), and referred to as “viral bulks/media” for simplicity, and (4) 95% (*v*/*v*) concentrated stabilizer solution (consisting of excipients; see Section 3) such that the target excipient levels were achieved after dilution with the 5% (*v*/*v*) viral bulks/media. Alterations in the 5% (*v*/*v*) viral bulk/media target volumes in certain experiments are described in the text.

#### 2.2.6. Lyophilization of Candidate Multidose MR Formulations

After preparing the candidate MR formulations described above, they were dispensed into sterile glass vials in a biosafety cabinet, and partially stoppered with rubber stoppers prior to lyophilization (and fully stoppered and crimped with aluminum seals after lyophilization). “Pre-lyo” vials were collected prior to lyophilization, and then crimped, weighed, and stored at −80 °C until analysis. A MR placebo formulation, containing 5% (*v*/*v*) culture media without virus (Minimum Essential Medium, MEM; Sigma, St. Louis, MO, USA) and 95% (*v*/*v*) stabilizer solution, was also prepared to fully load the lyophilizer as described below.

Lyophilization runs were performed using either a benchtop VirTis (SP Scientific, Warminster, PA, USA) [36] or pilot-scale LyoStar 2 or LyoStar 4 freeze-dryer (SP Scientific, PA, USA). Prior to each run, the freeze-dryer shelf was precooled to the appropriate temperature by using a standalone precool shelf cycle recipe (VirTis), or “Manual” (LyoStar 2) or “Semi-Automatic” (LyoStar 4) modes. Partially stoppered glass vials were transferred onto a freeze-dryer tray, with MR vaccine formulations placed in the center of the tray and MR placebo vials positioned around the tray perimeter. The full tray was placed onto the freeze-dryer shelf, and the tray bottom was removed to ensure direct vial contact with the shelf. Thermocouples were inserted into at least six vials. After sealing the chamber, the precooled shelf was turned off, and the “Freeze Drying” program was run in “Automatic” mode. Following cycle completion (using various temperature and pressure settings described in the text), the cabinet was backfilled with nitrogen and the glass vials were fully stoppered under vacuum (580 Torr) in the lyophilizer cabinet, then removed and crimped with aluminum seals.

The freeze-drying of various concentrations of the 5-dose candidate MR formulation (i.e., 1X, 1.5X, and 2X), filled into 4R glass vials, was initially performed using experimental lyophilization cycles prior to finalizing the final conservative cycle. These experimental freeze-drying cycles explored different freezing rates, primary drying, and secondary drying parameters to evaluate cake quality and viral titers. Compared with the final conservative cycles described next, many of these conditions were too aggressive and resulted in cake collapse. The final conservative lyophilization cycles used in this work were developed to ensure a prolonged, low-temperature freeze-drying process aimed at minimizing the risk of cake collapse and viral titer loss across various formulations, fill volumes, and vial types.

Briefly, different conservative lyophilization cycles were performed in the Lyo Star 2 unit (SP Scientific, PA, USA) to optimize shelf temperatures, pressures, and times during freezing, primary, and secondary drying. Slow freezing (2–8 °C shelf ramped at 1 °C/min to −55 °C) and a precooled shelf (−55 °C) were evaluated, and the completion of the primary drying cycle was determined by monitoring differential pressure between the Pirani gauge and the capacitance manometer [39]. Optimization of secondary drying cycles was performed to improve cake quality, viral titers, and residual moisture content as a function of different fill volumes in different glass vials. The optimized conservative cycle established for the 5-dose candidate MR formulation in the LyoStar 2 was then transferred to a LyoStar 4 freeze-dryer unit (SP Scientific, PA, USA). Initial runs displayed suboptimal cake quality, prompting evaluation of chamber pressure settings to reduce variability and improve outcomes. Modifications to primary drying temperatures and durations were carried out to optimize the lyo cycle, and the final conservative lyophilization cycle parameters selected for both the LyoStar 2 and LyoStar 4 freeze-dryers are described in Supplementary Table S1.

#### 2.2.7. Stability Studies with the Lyophilized Multidose Candidate MR Formulations

First, a short-term accelerated stability study (37 °C for 7 days) was performed using a single-dose, candidate MR formulation (±rHSA at 0.6 mg/mL), filled into 2R glass vials and lyophilized using the conservative lyophilization cycle (described in companion paper [36]) using the benchtop VirTis freeze-dryer (SP Scientific, PA, USA). The MeV and RuV viruses were formulated at 4.8 log TCID_50_/mL. The vials were filled with 0.65 mL of the candidate MR formulation and reconstituted using 0.6 mL WFI.

Next, real-time (2–8 °C) and accelerated (37 °C for 7 days) stability studies were performed on a five-dose, candidate MR formulation (at 2X concentration), filled into 4R glass vials, using the conservative lyophilization cycle developed for the LyoStar 4 freeze dryer (Supplementary Table S1). The candidate MR formulations were prepared at a target of 4.6 log TCID_50_/mL (4.3 log TCID_50_/mL post-reconstitution to 1X concentration) and lyophilized using the optimized LyoStar 4 conservative cycle described above. The effects of cell culture media volume (5 vs. 25% *v*/*v*) and the presence or absence of rHSA (0.6 mg/mL rHSA) were evaluated. Post-lyophilization assessments included cake appearance, reconstitution time, and residual moisture content. Viral titer losses were quantified using the PqPCR assay, comparing post-lyophilization samples to “pre-lyo” controls, and thermally stressed samples to “−80 °C” controls.

Two additional real-time stability studies (2–8 °C) were performed to support the above stability study using 1-dose candidate MR formulations (at 2X concentration), filled in 4R vials, lyophilized using the conservative lyophilization cycle developed for the benchtop VirTis freeze-dryer (SP Scientific, PA, USA) (described in our companion paper [36]). The first study used lower target MR titers of 4.6 TCID_50_/mL (4.3 log TCID_50_/mL post-reconstitution to 1X concentration), while the second study used a higher target MR titer of 5.1 TCID_50_/mL for each viral bulk (4.8 log TCID_50_/mL post-reconstitution to 1X concentration).

#### 2.2.8. MR Viral Infectivity Assays Using Potency qPCR (PqPCR)

A high-throughput duplex PqPCR infectivity-based potency assay (developed and implemented in our labs previously [36,40]) was used to quantify MeV and RuV log titers and titer losses in various MR formulations. Briefly, Vero cells (at 4 × 10^5^ cells/mL) were seeded into 96-well plates. The standard curve was generated by three-fold serial dilutions of freshly thawed MR bulks (corresponding to log-titers (TCID_50_/mL) 4.9–0.1 for MeV and 5.4–0.6 for RuV), and fifty microliters of each standard curve concentration, each MR formulation, or assay controls were added to wells containing 100 µL of cells in MEM + 2% (*v*/*v*) FBS. Infected cells were incubated at 35 °C and 5% CO_2_ for approximately 48 h. After incubation, culture medium was removed, wells were washed with DPBS, and 50 µL of 0.45% (*w*/*v*) Triton X-100 in nuclease-free water was used to lyse the cells. Samples were frozen at −80 °C for at least 1 h, then thawed in a biosafety cabinet, scraped thoroughly with a pipette tip, and agitated at 300 rpm at 2–8 °C for 20 min to ensure complete lysis. Cell lysates were diluted 20-fold in nuclease-free water, and the RT-qPCR reaction was set up as described in our companion paper [36]. Quantification was performed using a one-step duplex quantitative RT-PCR (referred to as potency qPCR or PqPCR in this report) with TaqMan^™^ 1-Step Multiplex Master Mix (Thermo Fisher, Waltham, MA, USA) with MR-specific primers [36,40].

#### 2.2.9. Osmolality Measurement

Osmolality values of the candidate multidose MR formulations were measured using 2X concentration lyophilized samples reconstituted to 1X by adding 3 mL WFI. Aliquots (30 μL) were transferred to sample tubes (Precision Systems Inc., Natick, MA, USA). Samples were analyzed using a 6002 Touch Micro Osmette Osmometer (Precision Systems Inc., Natick, MA, USA) in urine mode. The instrument was calibrated with 100, 500, and 1500 mOsm/kg standards and verified using a 290 mOsm/kg reference solution [36]. All measurements were performed in triplicate.

## 3. Results

**Figure 1:**
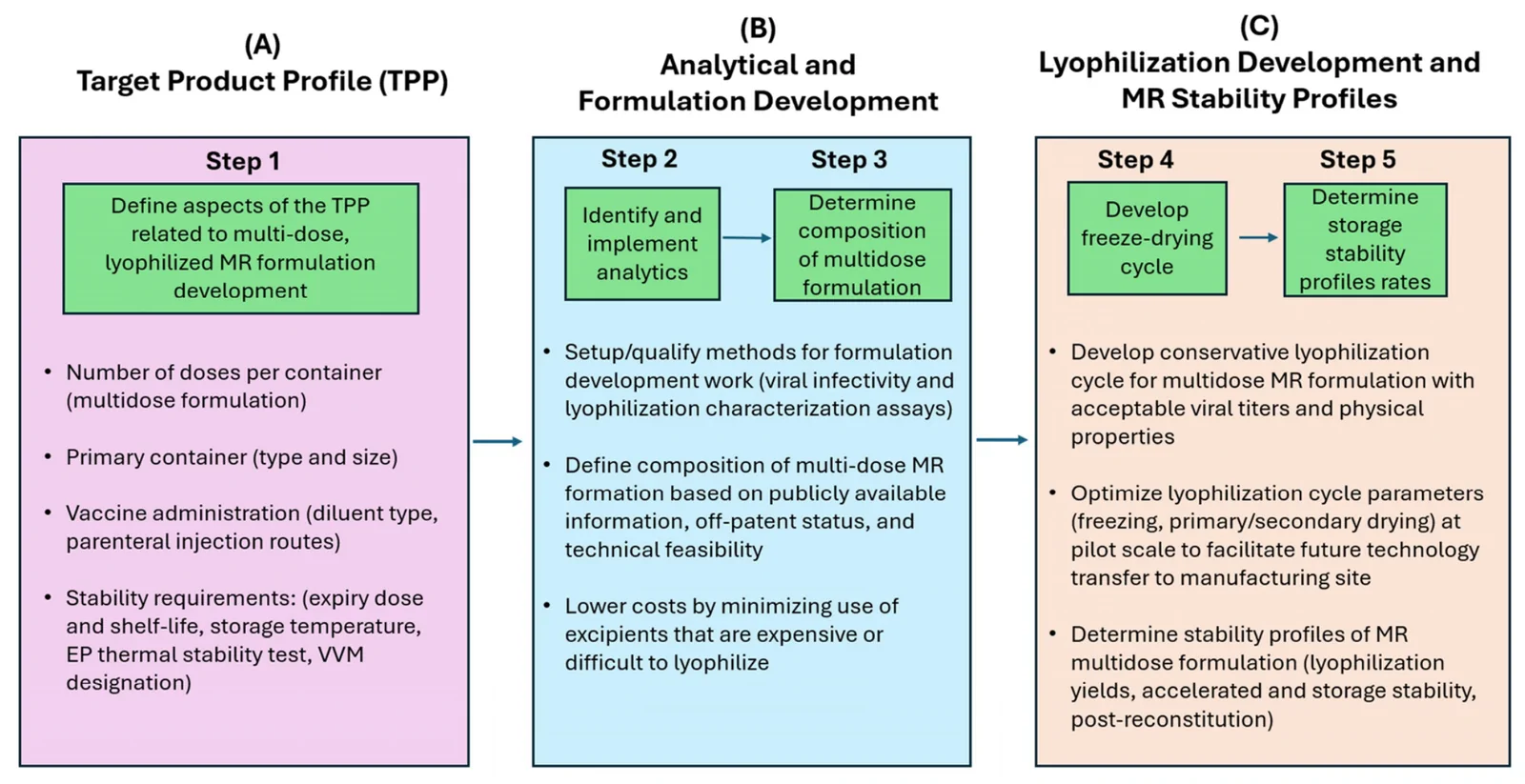
Overview of challenges encountered during formulation development of a stable, lyophilized, multidose presentation of an MR bivalent vaccine candidate. (**A**) Target product profile definition, (**B**) Analytical method establishment and formulation development, (**C**) Lyophilization cycle development and stability evaluation. This case study describes experimental work performed at lab-scale with the goal of future drug product scale-up, technology transfer, and local production at IPD in Senegal.

An overview of the challenges encountered during this case study to develop a stable, low-cost, lyophilized, multidose formulation of an MR vaccine candidate targeted for future drug product scale-up, technology transfer, and production in Africa at IPD, Senegal is illustrated schematically in Figure 1. This project required addressing three key challenges in a collaborative effort with consortium members (see Section 1). First, the team worked to define the formulation aspects of the MR vaccine candidate’s target product profile (TPP), including vaccine dosage form, administration, and stability requirements (Figure 1A). Next, a series of analytical (i.e., establishing analytical methods) and formulation development (defining the candidate MR formulation composition) topics were addressed (Figure 1B). Finally, lyophilization process development (i.e., optimizing the freeze-drying process and primary packaging for a multidose presentation) and drug product stability (establishing the MR stability profiles under real-time and accelerated conditions) were evaluated (Figure 1C). We present the experimental results generated to address these three key challenges in the subsequent sections.

### 3.1. Defining Formulation Development Goals in the Target Product Profile (TPP)

Overall, the TPP summarizes key goals of a vaccine candidate’s development (i.e., CMC, clinical, and regulatory), most of which are outside the scope of this formulation development work [35]. A TPP can be considered a “living document” that can be updated over time and covers a wide range of topics, including (1) potential clinical development strategies to achieve targeted levels of efficacy and safety [41,42], (2) vaccine distribution goals [43,44], (3) regulatory approval strategies for various regions and countries [45], and (4) production challenges including CMC process/product development goals, facilities, and manufacturing costs [46]. The TPP can also serve as a strong foundation to address other challenges associated with sustainable vaccine implementation, including financing strategies, training health care workers, and developing disease surveillance systems [45,47,48]. Thus, a TPP document provides a critical starting point to address multifaceted technical challenges that are encountered during development of a new MR vaccine candidate.

The aspects of the TPP focusing on goals for formulation development of a lyophilized, multidose MR vaccine candidate are highlighted in Figure 1A, with more details provided in Table 1, including (1) number of doses per vial, (2) vial type and size, (3) administration procedures, and (4) stability requirements. The vaccine dosage form was targeted to contain 5 doses, with the goal of vaccinating the maximum number of individuals while minimizing waste. As per the WHO guidance document [49], the 5-dose MR vaccine vial reduces wastage compared to the 10-dose vial (40% vs. 30%, respectively). Although the 5-dose vial costs more per dose (US$ 0.82 vs. US$ 0.66 for the 10-dose vial per the current maximum prices of Measles and MR vaccine accessible through UNICEF [49]), the overall cost also depends on wastage rates. For example, if the 5-dose vial’s wastage rate is ~26%, its cost per administered dose is comparable to the 10-dose vial’s cost, which has a ~40% wastage rate [49].

**Figure 2:**
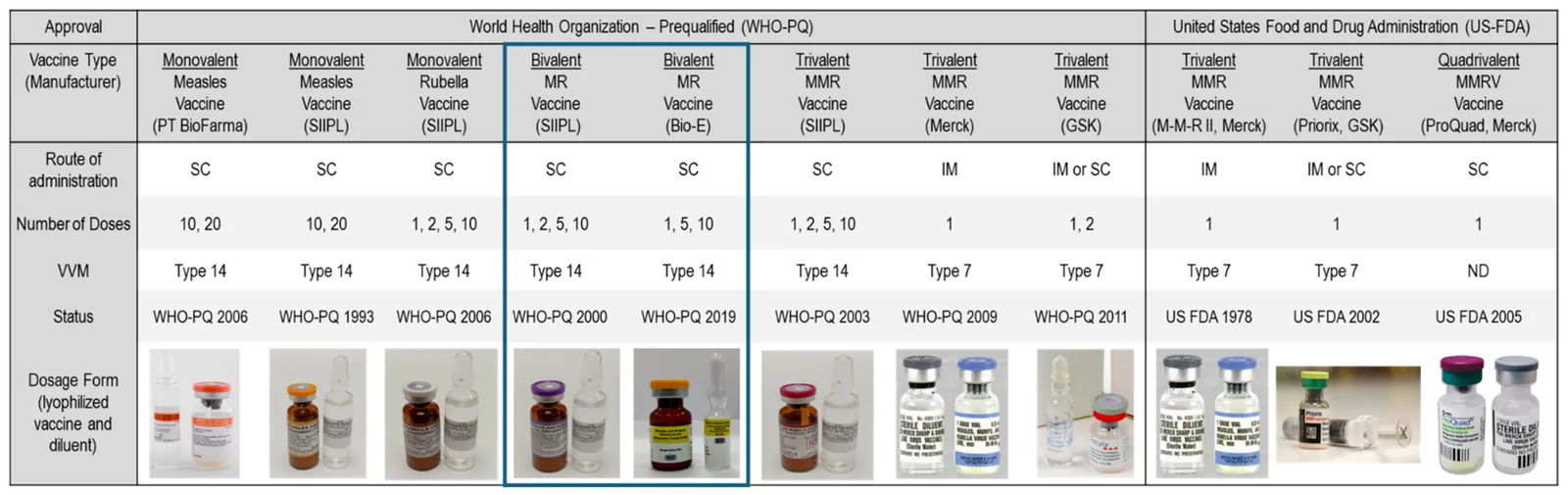
Examples of commercially available dosage forms of live, attenuated MeV and RuV-containing vaccines. Different compositions (monovalent, bivalent, trivalent, and quadrivalent combinations), configurations (primary containers, diluents), and regulatory status (VVM and approval dates) from WHO prequalification or US-FDA approval are shown. Bivalent MR vaccine dosage forms are highlighted in the box (in blue). VVM: vaccine vial monitor.

For this MR vaccine candidate’s primary packaging, a 4R clear pharmaceutical-grade glass vial was selected for the 5-dose presentation. The 4R vial size was selected based on numerous considerations including lyophilization throughput and costs [57], IPD manufacturing capabilities, and an evaluation of commercially available MeV and/or RuV containing vaccines. Figure 2 displays examples of commercial, MR-containing vaccines that have been prequalified by WHO or are approved by the US FDA, including monovalent, bivalent, trivalent, and quadrivalent combination vaccine formats [6]. Each presentation is lyophilized to retain MR vaccine potency and is manufactured in either amber or clear glass vials. The accompanying diluent to reconstitute the freeze-dried vaccine is supplied in either ampoules, vials, or prefilled syringes. Currently, there are two WHO-prequalified 5-dose MR bivalent vaccines (i.e., produced by Indian manufacturers SIIPL and Bio-E) (Figure 2, colored box) [7]. These two bivalent MR vaccines are lyophilized and supplied in amber-colored glass vials, accompanied by a diluent in separate ampoules. For the work described herein, the use of clear glass vials for the vaccine and the diluent was targeted, goals which align with other US FDA-approved trivalent and quadrivalent MR-containing vaccines including GSK’s MMR (PRIORIX^®^), as well as Merck’s MMR (M-M-R^®^II) and MMRV (ProQuad^®^) [6]. Similar to these examples, the candidate MR vaccine in this work will require protection from light exposure, which is known to cause MeV and RuV inactivation [58,59], by using appropriately opaque secondary packaging.

The TPP targets for vaccine administration and stability goals are summarized in Table 1. The expiry dose of the MR bivalent vaccine candidate (after reconstitution and same-day storage) is targeted at 3.0 log TCID_50_/dose for each virus, which aligns with the minimum dose reported to be used in most commercial MR-containing vaccines (both MeV and RuV at 3.0 log TCID_50_/dose) [6,7,30]. Some commercial MR-containing vaccines, however, have higher minimum expiry doses with MeV at 3.4 and RuV at 3.3 log TCID_50_/dose [6,7,60]. The reconstituted MR vaccine candidate in this work is targeted for IM or SC administration depending on the design of future clinical trials. In terms of storage stability, the MR vaccine candidate is targeted for up to two years of storage at 2–8 °C. Under accelerated stability conditions (37 °C for 7 days), it is expected to meet the European Pharmacopoeia (EP) thermostability test criteria for MeV [53] and trivalent MMR [54], i.e., no more than a 1 log reduction in potency loss. Following reconstitution, the MR vaccine candidate is targeted to retain its minimum effective dose for up to 8 h at 2–8 °C prior to administration. The Vaccine Vial Monitor (VVM) designation is targeted as Type 14, the same VVM type listed by the two WHO-prequalified MR bivalent vaccines [7].

### 3.2. Setting up Key Analytical Methods for MR Vaccine Formulation Development Work

The first set of technical challenges encountered in this work was defining the analytical methods (Figure 1B). To this end, a high-throughput reverse transcription quantitative polymerase chain reaction (RT-qPCR)-based viral infectivity assay was used for potency measurements (PqPCR), as described previously [40]. This PqPCR was implemented as a surrogate assay to measure MeV and RuV viral titers for formulation development, and has been previously correlated to the commonly used quality control (QC) potency assay (Tissue Culture Infectious Dose 50%, TCID_50_) in terms of monitoring MeV and RuV stability profiles [40]. Key benefits of using the PqPCR assay include the use of a single cell type (Vero cells) for both MeV and RuV infections in the duplex format, enabling simultaneous analysis of both viruses in the combined drug product without the need for anti-Rubella antiserum. In this work, the PqPCR assay was first used to evaluate the concentrated viral bulks and was shown to display similar assay performance as described previously with different MR bulks [40]. For example, the infectivity titers of the monovalent and bivalent unformulated viral bulks, formulated viral bulks, and reconstituted candidate MR formulation were determined with an assay variability of ±0.2 and 0.3 log TCID_50_/mL for Me and RuV, respectively.

Additional assays were also set up to enable MR vaccine formulation development, including analyses of (see Section 2.2): (1) the lyophilized formulation (i.e., physical cake quality, moisture content, glass transition temperature, Tg, and reconstitution time), and (2) the liquid version of the MR formulation either before lyophilization or after reconstitution) (i.e., glass transition temperature (*T_g_*′) of the maximally freeze-concentrated liquid, osmolality measurements).

### 3.3. Defining the Composition of a Candidate Multidose MR Vaccine Formulation

The composition of the candidate multidose MR vaccine formulation with acceptable stability profiles to fulfill the TPP goals was determined based on various factors including (1) availability and composition of the MR bulks, (2) use of lower-cost excipients that are readily available (i.e., GMP quality) that can be administered parenterally, and (3) anticipated ability to be lyophilized at large scale in a multidose configuration (i.e., larger fill volumes in larger glass vials). As a starting point, the composition of the off-patent, commercially available, single-dose, lyophilized M-M-R^®^II vaccine was evaluated using publicly available information from several different sources, including package inserts [52] a product monograph [30] and expired patents and patent applications [37,61,62,63]. Several modifications to this single-dose, trivalent M-M-R^®^II formulation composition were evaluated and implemented to achieve the TPP goals outlined above for developing a candidate multidose MR formulation for production in Africa. These adaptations were separated into three categories: (i) excipients and tissue culture media components (Table 2; see category A: Excipients), (ii) process residuals derived from the production of the MeV and RuV bulks (Table 2; see category B: Manufacturing process residuals), and (iii) the vaccine strains and viral infectivity titers of the MeV and RuV bulks (Table 2; see category C: Viral bulks).

The first category (Table 2; see category A: Excipients) summarizes excipients that are common and different between the two formulations, including: polyols/carbohydrates (sorbitol and sucrose), protein (hydrolyzed gelatin), phosphate buffer components (mono- and dibasic sodium and potassium phosphate), and other additives (sodium glutamate and sodium bicarbonate). The combination of sorbitol and gelatin is a well-known stabilizer of live viral vaccines including MeV and RuV [40,64,65], while the other excipients were likely added to improve viral stability either as part of viral bulk production or during freeze-drying [25]. Since this category also includes compounds that are derived from the tissue culture media used to produce and dilute the viral bulks to the target titer, differences are expected since different culture media have varying compositions (e.g., M199 vs. MEM) at different concentrations (e.g., less volume required when using the concentrated viral bulks).

For the second category (Table 2; see category B: Manufacturing process residuals) of manufacturing residuals, differences are also expected between the two formulations since the concentrated MeV and RuV bulks utilized in this work are made with different cell substrates as well as different manufacturing processes (i.e., upstream and downstream processing steps). The most notable component in this category is the rHSA, both in terms of its potential effects on viral stability and vaccine manufacturing costs; therefore, the role of rHSA was examined in more detail as part of formulation development of the candidate MR multidose formulation (see below).

For the third category (Table 2; see category C: Viral bulks) of vaccine strains and viral titers, the candidate MR vaccine formulation in this work excludes Mumps due to epidemiological, financial, and logistical reasons, since MeV and RuV pose a much higher public health risk than Mumps in AFRO [66]. Additionally, the MR vaccine is less expensive and easier to manage within limited healthcare budgets and infrastructure supported by local governments and global health initiatives, making bivalent MR the more practical and cost-effective choice for the region [67]. The MeV vaccine strains used in the two formulations are different and grown in different cell substrates (Edmonston–Zagreb strain grown in MRC-5 cells in this work vs. Enders–Edmonston strain grown in chick embryo cells). For RuV, the same vaccine strain is used (Wistar RA 27/3) but grown in different host cell lines (MRC-5 cells in this work vs. WI-38 cells). From a formulation perspective, such differences may potentially affect the stability profiles of the live, attenuated viruses [25]. For example, it has been reported that MeV grown in different cell lines (and thus containing different viral envelope lipid compositions) or under different cell-culture conditions can display different stability profiles [68]. Finally, to achieve the target viral infectivity titer values, it has been reported that 25% of the total volume is reserved for the three viral bulks used to produce the M-M-R^®^II, with the remaining 75% volume comprising the stabilizer solution [37,63]. In contrast, by utilizing concentrated MeV and RuV bulks in this work, the candidate MR multidose formulation was prepared with 5% of the total volume reserved for viral bulks/culture media, leaving the remaining 95% of volume for the stabilizer solution. The effect of volume percent of viral bulks/culture media on lyophilization performance was examined in this work as described in more detail below.

**Table 2:** Estimated composition (per dose) of candidate bivalent, multidose MR formulation developed in this case study compared to reported composition of trivalent, single-dose M-M-R-^®^-II vaccine. Differences are highlighted in graey color. * Composition of M-M-R-^®^-II vaccine based on publicly available information [30,37,38,52]. ** Calculations for the candidate MR multidose formulation based on results from one lot of each of a MeV and RuV bulks (see Section 2.1).

Category	Ingredient	Reported M-M-R^®^-II Composition * (Quantity/0.5 mL Dose After Reconstitution)	Estimated Composition of Candidate MR Multidose Formulation in this Work ** (Quantity/0.5 mL Dose After Reconstitution)	Comments on the Candidate MR Multidose Formulation
**A: Excipients**	Sorbitol	14.5 mg		Same excipients and targeted amounts
	Sucrose	1.9 mg		
	Hydrolyzed gelatin	14.5 mg		
	Sodium phosphate monobasic	3.1 mg		
	Sodium phosphate dibasic (anhydrous)	2.2 mg		
	Potassium phosphate dibasic (anhydrous)	30 µg		
	Potassium phosphate monobasic	20 µg		
	Sodium bicarbonate	0.5 mg		
	Monosodium L-glutamate monohydrate	20 µg		
	Medium 199 with Hank’s salts	3.3 mg	--	Differences due to use of conc MeV and RuV bulks (made with DMEM/MEM media without neomycin)
	Minimum Essential Medium, Eagle	0.1 mg	To volume 5% *v*/*v*	
	Neomycin	25 µg	---	
	Phenol Red	3.4 µg	0.0003–0.0005 µg	
	Water for injection	To volume		
**B: Manufacturing process residuals**	Recombinant human albumin (rHSA)	≤0.3 mg	0.002–0.005 mg	Differences due to rHSA excluded from conc MeV bulks and trace amounts from conc RuV bulks
	Fetal bovine serum (FBS)	<1 ppm	<1 ppm	Regulatory requirement [69]
**C: Viral bulks**	Measles virus	Enders’ Edmonston strain (grown in chick embryo cells in M199)	Edmonston–Zagreb strain (grown in human MRC-5 cells in DMEM)	Different MeV strain grown in different cells in different culture media
	Rubella virus	Wistar RA 27/3 strain (grown in human WI-38 cells in MEM)	Wistar RA 27/3 strain (grown in human MRC-5 cells in MEM)	Same RuV strain grown in different cells in same culture media
	Mumps virus	Jeryl Lynn^®^ (B level) strain (grown in chick embryo cells in M199)	--	Excluded
	Percent formulation volume: diluted viral bulk (VB) and stabilizer solution (SS)	VB: Est 25% (*v*/*v*) SS: Est 75% (*v*/*v*)	VB: 5% (*v*/*v*) SS: 95% (*v*/*v*)	Differences due to use of conc MeV and RuV bulks

### 3.4. Reducing rHSA Levels and Viral Bulk Volumes to Lower Formulation Costs

Based on the vaccine composition considerations outlined above, we identified two important parameters for the candidate multidose MR formulation to lower costs: (1) minimizing the amount of rHSA in the stabilizer solution, and (2) reducing the volumes of viral bulks to improve ability to freeze-dry at large scale. The rHSA present in commercial M-M-R^®^II formulation is reported as a manufacturing process residual (i.e., Recombumin, produced in yeast, up to 300 mcg/dose or 0.6 mg/mL) [70]. Thus, rHSA likely comes from the viral bulk manufacturing process and is subsequently carried over into the final dosage form. Therefore, its effect on lyophilization yields and storage stability is difficult to evaluate since the viral bulks contain rHSA prior to addition of the stabilizer solution. In this work, with the availability of concentrated, higher-titer MeV and RuV bulks produced with none to minimal rHSA (i.e., the MeV bulks lacked rHSA and RuV bulks were greatly diluted, resulting in minimal carryover to the final vaccine formulation estimated at 2–5 mcg/dose), we had the opportunity to evaluate the effect of rHSA in the stabilizer solution on MeV and RuV stability profiles of the lyophilized candidate MR formulation.

**Figure 3:**
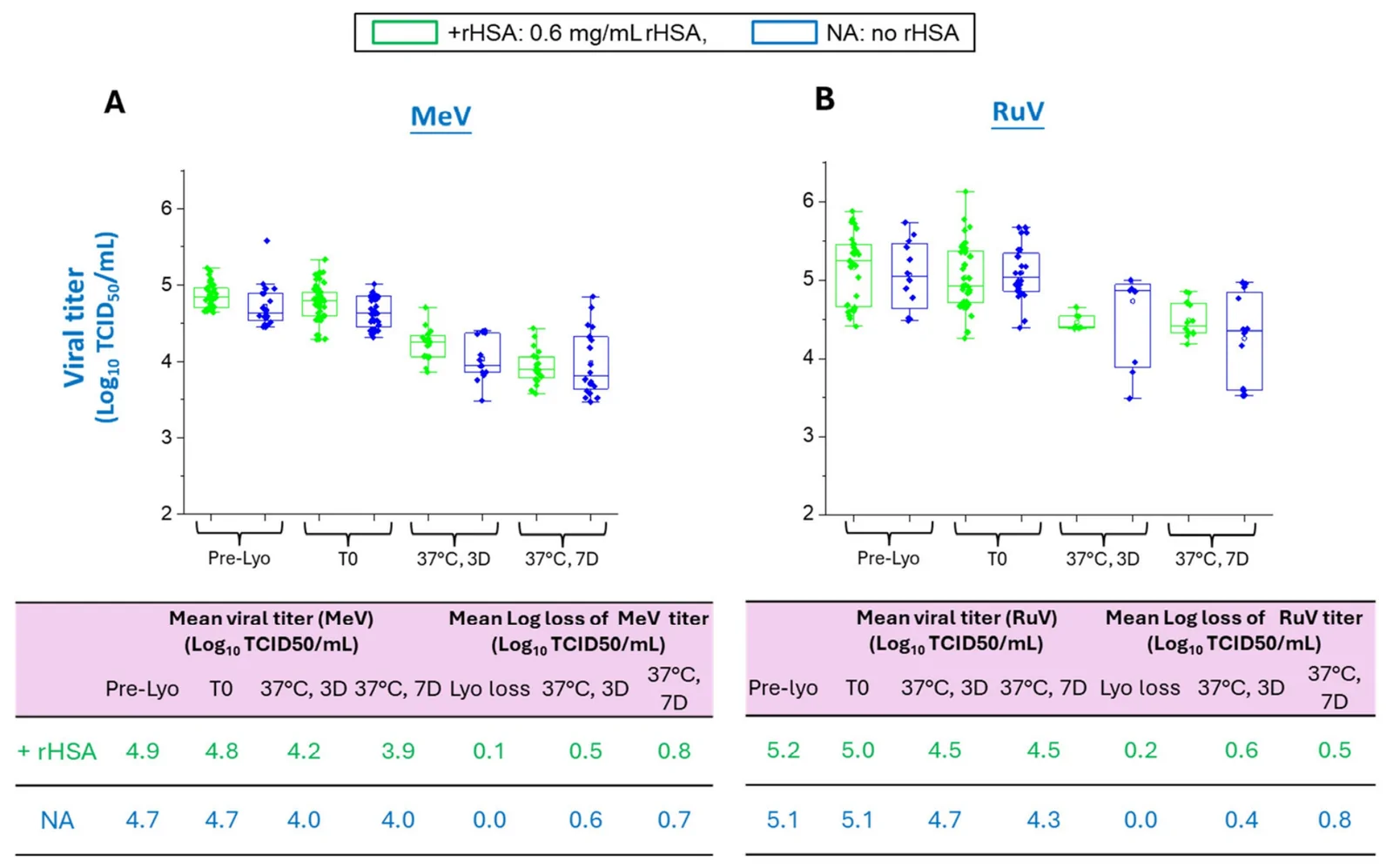
Effect of absence vs. presence of recombinant human serum albumin (rHSA) in the stabilizer solution used to prepare the candidate MR formulation on lyophilization yields and accelerated stability profiles of (**A**) MeV and (**B**) RuV. The candidate MR formulation was prepared at 5% (*v*/*v*) MR bulks/culture media and 95% (*v*/*v*) stabilizer solution and filled as a single-dose volume (0.65 mL) and lyophilized in 2R glass vials using a lab-scale VirTis freeze-dryer. Viral log10 titers were measured by potency qPCR. Data across four independent experiments are displayed as box-and-whisker plots (*n* = 16–56 for MeV; *n* = 8–40 for RuV). See Table 2 for the estimated composition of the candidate MR formulation.

To this end, we prepared two candidate MR formulations, i.e., 5% *v*/*v* viral bulks/culture media combined with 95% *v*/*v* stabilizer solution, the latter prepared with and without rHSA (i.e., Exbumin produced in rice at the higher-end of levels reported in M-M-R^®^II, 0.6 mg/mL or 300 mcg/dose). The two candidate MR formulations were filled at single-dose volume (0.65 mL) into 2R glass vials and lyophilized using a lab-scale, benchtop lyophilizer (using a conservative lyo cycle as described below and [36]). Across four independent experiments, no notable effect of rHSA (0.6 mg/mL) in the candidate MR formulation was observed after formulation, lyophilization, and thermal stress (37 °C for 3 and 7 days) for both MeV (Figure 3A) and RuV (Figure 3B) as measured by the potency qPCR assay. All formulations met the criteria of EP thermal stability testing (not more than one log loss after 1 week at 37 °C), and similar trends were observed at the earlier 3-day timepoint. Also, no differences between candidate MR formulations ± rHSA were observed in the physical properties of the lyophilized cakes, including appearance, residual moisture, and reconstitution time. Based on these results, rHSA is not required to maintain virus stability as part of the stabilizer solution used to prepare the candidate multidose MR vaccine formulation, offering a significant opportunity to lower costs.

**Figure 4:**
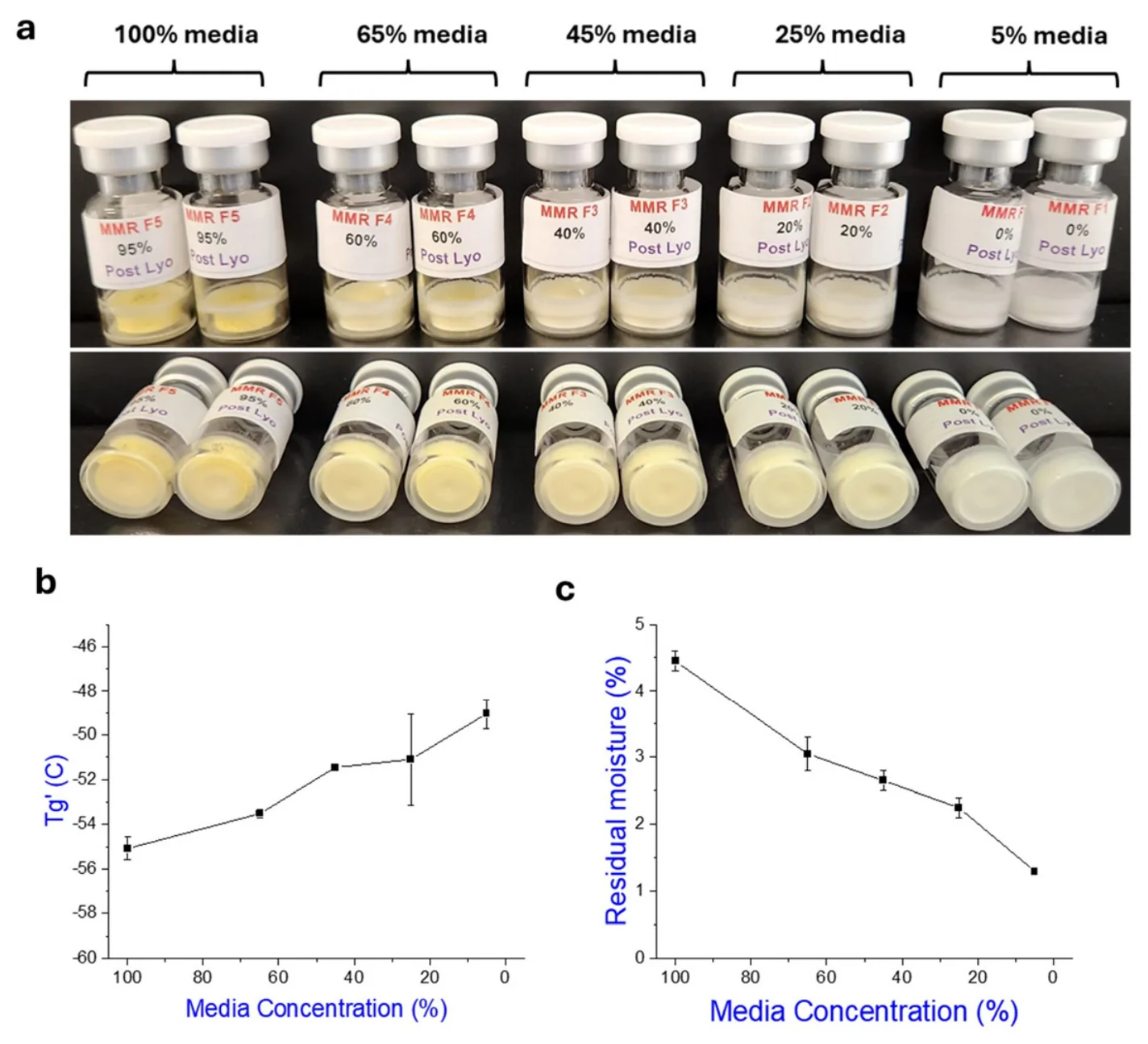
Effect of volume ratio of cell-culture media to stabilizer solution on the lyophilization performance of the candidate MR formulation. (**a**) representative vials showing appearance of the lyophilized cakes (single-dose fill volume 0.65 mL filled into 2R vials), (**b**) thermal glass transition value (*T_g_*′) of maximally freeze-concentrated form of the formulations as measured by mDSC, and (**c**) residual moisture content after freeze-drying as measured by the Karl Fischer titration. The *T_g_*′ and residual moisture values are presented as the mean ± range (2 vials, each vial analyzed at *n* = 1). See Table 2 for the estimated composition of the candidate MR formulation at the 5% (*v*/*v*) viral bulks/media and 95% (*v*/*v*) stabilizer solution.

Regarding the volumes of viral bulks used in the candidate MR formulation, this range will ultimately depend on the viral infectivity titer values of the MeV and RuV bulks from future large-scale production batches (i.e., higher bulk titers result in lower volumes added to the formulation). To keep the volume ratios of bulks to stabilizer solution consistent from batch-to-batch, a single ratio should be selected which accommodates the lowest titer bulks and allows for a dilution of the higher titer bulks. We therefore performed a study that titrated the volume of culture media (i.e., 5, 25, 45, 65, and 100% (*v*/*v*)) and the corresponding inverse volume of stabilizer solution to assess its impact on the cake quality and MeV and RuV stability. The study was conducted using the candidate MR formulation (+rHSA at 0.6 mg/mL) that was filled and lyophilized (2R vials, LyoStar 2, using a conservative lyo cycle). The results demonstrated that higher culture media concentration led to poorer cake quality (Figure 4a), lower *T_g_*′ values (Figure 4b), and higher residual moisture (Figure 4c). Interestingly, no major effect on viral infectivity titers in terms of lyophilization yields and thermal stability was observed in the range of 5 to 45% (*v*/*v*) culture media (data not shown). Based on these results, the lowest viral bulk volume (5% *v*/*v*) was selected as the target volume in the candidate MR formulation, a result offering significant opportunity to improve performance during freeze-drying of a multidose formulation.

### 3.5. Lyophilization Development for the Candidate Multidose MR Formulation

An overview of the lyophilization development workflow followed in this study is shown in Figure 1C. The lyophilization process involves three key stages: freezing, primary drying, and secondary drying. During freezing, the shelf temperature is lowered below the *T_g_*′ value of the formulation (i.e., the glass transition temperature of the maximally freeze-concentrated phase), leading to ice crystal formation of the bulk water and concomitant solute concentration [71]. This is followed by primary drying, during which reduced chamber pressure and controlled heat input drive sublimation of ice to remove bulk water [32]. Finally, secondary drying removes residual bound water through desorption at elevated shelf temperatures, which is characterized by determining the *T_g_* value (glass transition temperature of the final dried cake) [39].

As a first step to develop a lyophilization cycle, the *T_g_*′ and *T_g_* values for the candidate MR formulation (at 5% and 95% *v*/*v* of virus bulk/media and stabilizer solution, respectively) were determined. The *T_g_*′ value was measured by mDSC as approx. −52 °C. Thus, a very cold shelf temperature (and therefore slow sublimation rates resulting in long drying times) is required to prevent cake collapse during sublimation. Since *T_g_*′ of this formulation varied as a function of the volume percent of viral bulks/media in the formulation (shown above), the smallest volume percent (5% *v*/*v*) is most compatible with the freeze-drying process. This result is not unexpected given the low *T_g_*′ values of the key components of the formulation, including salts and sorbitol [30,72]. Next, the *T_g_* value of the dried cake was determined by mDSC as approx. 45 °C. This value is the highest temperature exposure at which the solid cake will remain intact during the freeze-drying process and subsequent storage. Based on the *T_g_*′ and *T_g_* properties of the candidate MR formulation, a conservative lyophilization cycle (i.e., 4 days total time using a primary drying shelf temperature of −55 °C and a secondary drying shelf temperature in the range of −15 to 30 °C) was developed and used to perform the initial candidate MR stability results described above.

**Figure 5:**
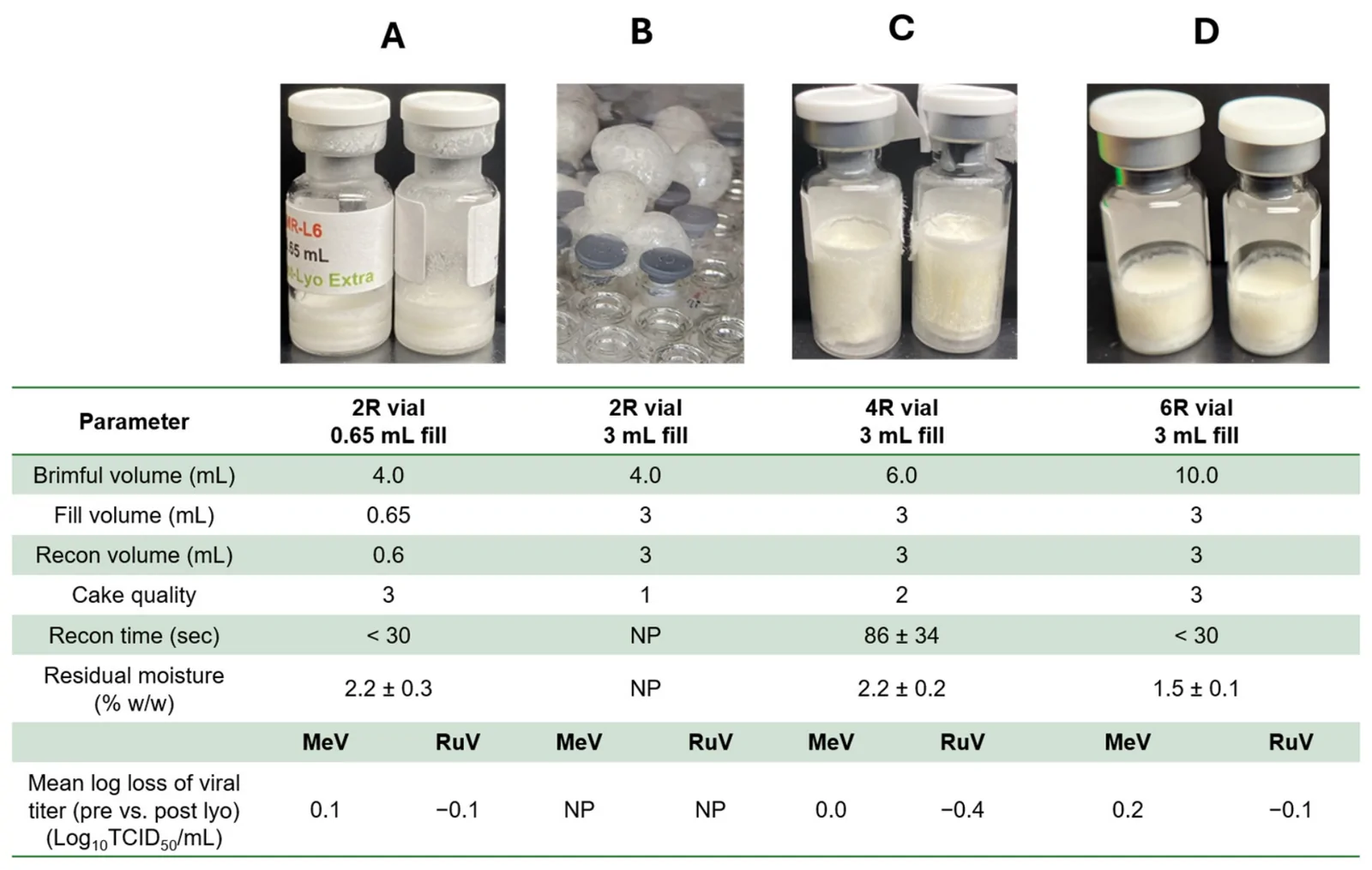
Lyophilization of the candidate MR formulation filled into glass vials of different sizes. Results of cake quality, reconstitution time, residual moisture levels, and lyophilization yields (viral infectivity titer losses for MeV and RuV by potency qPCR assay) are shown for (**A**) 2R vial with single-dose fill volume, (**B**) 2R vial with a 5-dose fill volume, (**C**) 4R vial with a 5-dose fill volume, and (**D**) 6R vial with a 5-dose fill volume. The samples were produced using intermediate lyophilization cycles prior to finalizing the conservative lyophilization cycle (see Section 2). The cake quality was assessed by visual observation using a scale of 3 = excellent, 2 = good-to-fair, and 1 = poor. Log loss of viral titer data are presented as the mean values (2 vials, each vial analyzed at *n* = 4 with SD range of 0.1 to 0.3). See Table 2 for the estimated composition of the candidate MR formulation. NP—not performed.

To develop a multidose formulation, we first evaluated the various vial sizes (i.e., 2R, 4R and 6R) for a 5-dose candidate MR formulation (i.e., 3 mL total fill volume to account for five doses of 0.5 mL each and an overfill to ensure all five doses can be recovered). We lyophilized the candidate multidose MR formulation (containing rHSA) in different vials, as well as a control single-dose configuration, using the conservative lyo cycle. Pictures of lyo cake qualities in the different vial sizes and obtained experimental results are presented in Figure 5A–D. In the control samples (single dose volume of 0.65 mL in a 2R vial), good cake quality and reconstitution time (<30 s) were observed along with an acceptable moisture content of 2.2 ± 0.3% (*w*/*w*). None to minimal MeV and RuV infectivity titer losses were observed post-lyophilization. These results were then compared to the 3.0 mL 5-dose fill volume of the same candidate MR formulation filled into 2R, 4R, and 6R vials (brimful volume of ~4, 6, and ~10 mL, respectively) [73].

For the 2R vial with the 3.0 mL fill volume, results indicated incomplete primary drying, with some cakes completely collapsed and others showing material “bubbling out”; therefore, the 2R vial configuration was not considered further since it cannot accommodate a 5-dose fill volume. For the 4R vial with the 3.0 mL fill volume, results showed good-to-fair cake quality with recon times and residual moisture contents within reasonable limits. Zero to minimal titer losses for both viruses were observed after lyophilization. For the 6R vial with the 3.0 mL fill volume, excellent cake quality along with reasonable recon times and residual moisture levels were observed. Zero to minimal titer losses for both viruses were observed after lyophilization. Considering these data, along with the TPP profile, the 4R glass vials were selected for further evaluation.

**Figure 6:**
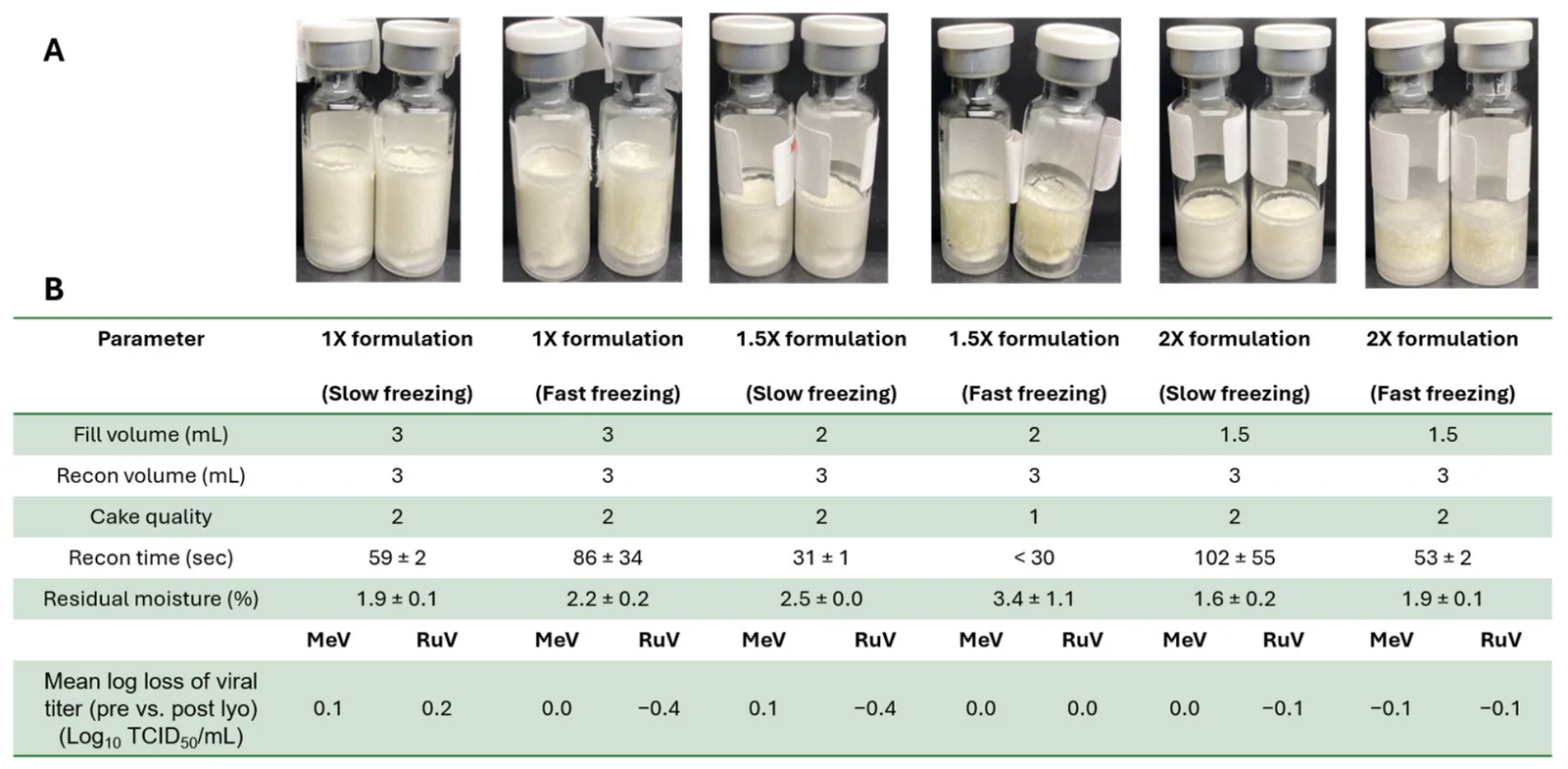
Lyophilization of different concentrations and fill volumes of the 5-dose candidate MR formulation in 4R glass vials. (**A**) representative vials displaying lyophilized cake quality appearance from slow and fast freezing cycles from various concentrations of the candidate MR formulation, and (**B**) fill and reconstitution volumes along with experimentally measured parameters of the lyophilized and reconstituted samples. The samples were produced using intermediate lyophilization cycles prior to finalizing the conservative lyophilization cycle (see Section 2). Cake quality was assessed by visual observation using a scale of 3 = excellent, 2 = good-to-fair, and 1 = poor. Log loss of viral titer data are presented as the mean values (2 vials, each vial analyzed at *n* = 4 with SD range of 0.1 to 0.4). See Table 2 for the estimated composition of the candidate MR formulation.

The relatively high fill volume height resulting from adding 3.0 mL of the candidate MR formulation into 4R vials introduced potential future challenges related to longer sublimation times and potentially retaining container-closure integrity (i.e., avoiding any spillage of sample from the vial). Therefore, we focused next on improving the lyophilization process in a series of experiments varying lyophilization parameters such as shelf temperature, time, and pressure settings. We further evaluated varying concentrations (i.e., 1X, 1.5X, and 2X) of the candidate MR formulation (in the presence of rHSA), leading to smaller fill volumes (i.e., 3 mL, 2 mL, and 1.5 mL) in 4R vials (while reconstituting the lyophilized vials with an appropriate amount of diluent to achieve 3.0 mL, 1X formulation concentration). In addition, we evaluated slow-freezing (2–8 °C shelf ramped at 1 °C/min to −55 °C) and fast-freezing (using a precooled shelf at −55 °C). Overall cake qualities for 1X, 1.5X, and 2X formulation samples were good-to-fair independent of freezing rate, except for fast freeze,1.5X formulation, which was poor (Figure 6A). The experimental results for reconstitution time and residual moisture were within similar ranges, and lyophilization yields (pre- vs. post-lyo viral infectivity titers) showed zero to minimal titer losses for both viruses (Figure 6B). We selected a 2X concentration candidate MR formulation (1.5 mL fill volume) and a fast-freezing rate to (1) minimize primary drying times by sublimating the lowest bulk water volume, (2) avoid any potential for spillage during lyophilization by using the lowest fill volume, and (3) fast freezing is conceptually preferred to improve (compared to slow freezing) both freeze-drying efficiency [74] and the stability of enveloped live viral vaccines [25].

### 3.6. Conservative Lyophilization Cycles in Pilot-Scale Freeze-Dryers for the 5-Dose, 2X Concentration Candidate MR Formulation Filled into 4R Vials

The conservative lyophilization cycle for the 5-dose, 2X concentrated candidate MR formulation (with 1.5 mL filled into 4R vials) is an 8-day freeze-drying process. When using two different pilot-scale freeze-dryers (LyoStar 2 and LyoStar 4), however, different parameters were required to optimize the drying process to achieve the same total cycle time. The LyoStar 2 freeze-dryer was a legacy instrument that required repair at this stage of the project (with technical support no longer available from the manufacturer). We then obtained a new and updated pilot-scale unit, the LyoStar 4, which was successfully set up and evaluated. We observed that the conservative lyophilization cycle parameters established on the LyoStar 2 did not work as expected on LyoStar 4. This result required further changes to the lyophilization process parameters and was likely attributed to differences in vacuum pump configuration (the LyoStar 2 was equipped with an oil pump whereas the LyoStar 4 is equipped with a dry scroll pump), which affected primary drying performance for a low *T_g_*′ formulation.

**Figure 7:**
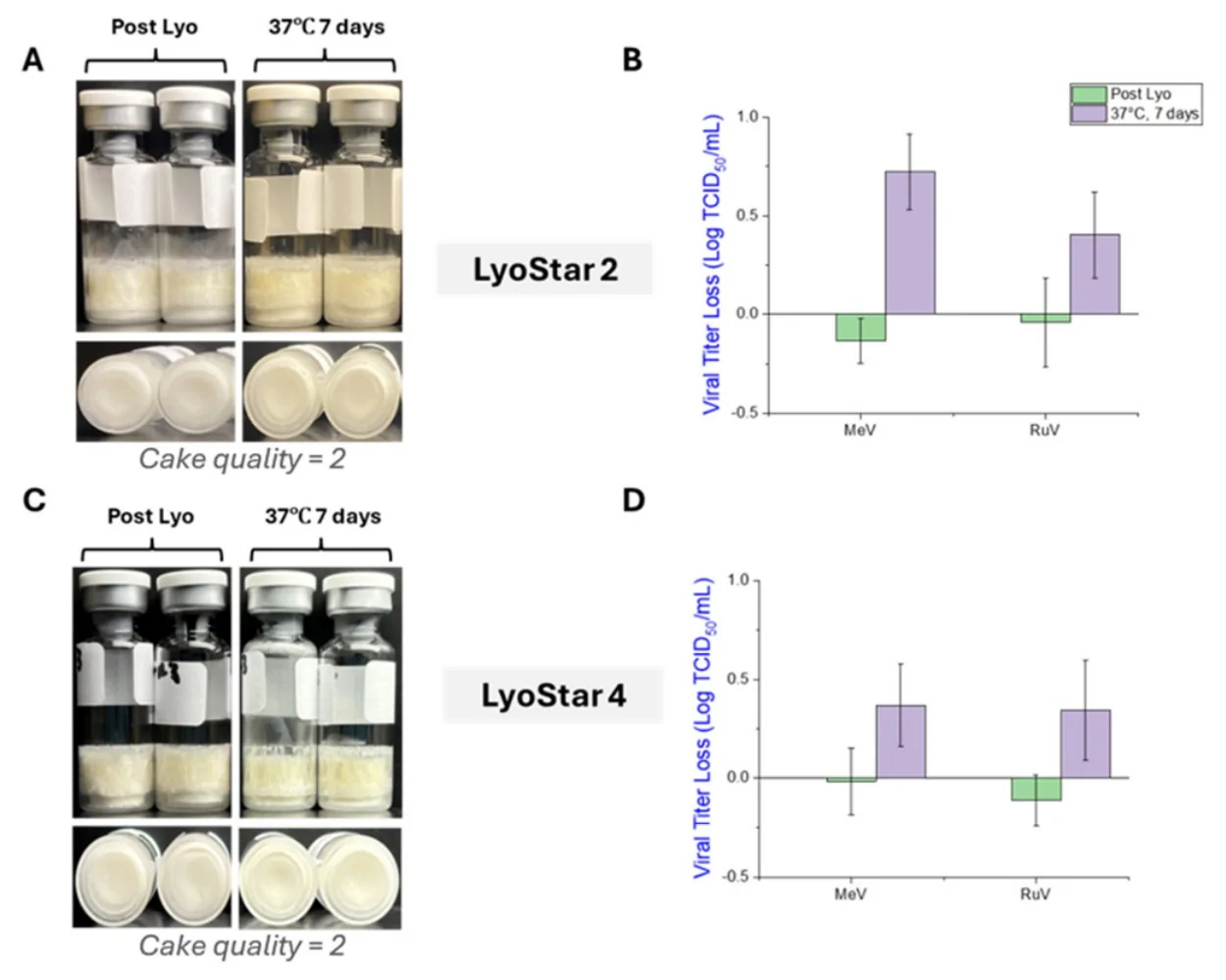
Comparison of the 5-dose 2X concentrated candidate MR formulation filled into 4R vials after lyophilization in two different pilot-scale freeze-dryers (LyoStar 2 vs. LyoStar 4). Samples were lyophilized using their respective optimized conservative lyo cycles for each unit. Samples were evaluated after lyophilization and following thermal stress at 37 °C for 7 days. For the LyoStar 2, (**A**) cake quality and (**B**) log loss of MeV and RuV infectivity titers as measured by PqPCR. For the LyoStar 4, (**C**) cake quality and (**D**) log loss of MeV and RuV infectivity titers (vs. unstressed −80 °C control). Viral titer loss data are presented as the mean ± 1 SD (2 vials, each vial analyzed at *n* = 4). The cake quality was assessed by visual observation using a scale of 3 = excellent, 2 = good-to-fair, and 1 = poor. See Table 2 for the estimated composition of the candidate MR formulation.

A comparison of the 5-dose, 2X concentrated candidate MR formulation, filled into 4R vials and freeze-dried in the two different pilot-scale lyophilizers, is provided in Figure 7 (i.e., 2X concentrated MR formulation containing rHSA, with a fill volume of 1.5 mL, and reconstituted with 3.0 mL WFI resulting in targeting 4.3–4.8 log TCID_50_/mL of each virus at a final 1X concentration of the formulation). For the LyoStar 2, differential pressure measurements (Pirani gauge vs. CM) indicated that primary drying concluded after ~140 h. Post-lyophilization, cake qualities were good-to-fair (Figure 7A), and the residual moisture content was 2.3 ± 0.3%. For viral stability, no losses were observed in either MeV or RuV infectivity titers pre- vs. post-lyophilization. After storage for 7 days at 37 °C, MeV and RuV titer losses were <1.0 log (i.e., MeV: 0.7 log TCID_50_/mL; RuV: 0.4 log TCID_50_/mL) (Figure 7B). With respect to the LyoStar 4, differential pressure measurements (Pirani gauge vs. CM) indicated that primary drying concluded at ~125 h. Post-lyophilization, cake qualities were good-to-fair (Figure 7C), and the residual moisture content was 2.4 ± 0.3%. For viral stability, no losses were observed in viral infectivity titers pre- vs. post-lyophilization. After storage for 7 days at 37 °C, MeV and RuV titer losses were <1.0 log (i.e., MeV: 0.4 log TCID_50_/mL; RuV: 0.4 log TCID_50_/mL) (Figure 7D).

The conservative lyophilization cycle described had a total lyophilization time of 8 days, which is a very long drying process and would be a challenge in terms of productivity within a large-scale vaccine manufacturing facility (e.g., ideally, 2–5 days would be preferred, per personal communications). As an initial step to address this limitation, a series of cycle optimization studies were performed using the LyoStar 4 unit with the 5-dose, 2X concentration, candidate MR formulation filled into 4R vials. Two promising lyophilization cycles were identified that shortened the total lyophilization time to ~7 or ~6 days, respectively (see first and second tech-transfer lyophilization cycle parameters in Supplementary Table S2). This Initial proof-of-concept was established by examining cake quality, residual moisture, reconstitution time, and viral titer loss after lyophilization and thermal stress (7 days at 37 °C) (data not shown). These initial results can be used as a starting point for future technology transfer studies to further shorten the total lyophilization cycle time.

### 3.7. Stability Profiles of the 5-Dose, 2X Concentrated Candidate MR Formulation

A series of three separate real-time (2–8 °C) and accelerated temperature storage stability studies were performed to evaluate the stability profile of MeV and RuV in four different versions of the 5-dose, 2X concentrated candidate MR formulation filled into 4R vials. The four variations included differences in culture media composition (5% vs. 20% *v*/*v*) and presence and absence of rHSA in the stabilizer solution (0.6 mg/mL final concentration). In addition to rank-ordering the four variations of the formulation, the stability profile trends were used to estimate the release dose of the MeV and RuV components required to ensure minimum potency at expiry. This “vaccine stability window” spans from the initial release dose (titer values to account for losses due to formulation, filling, and lyophilization, as well as assay variability), down to the expiry shelf-life date that refers to the storage time at which the lowest clinically effective dose is reached (value accounts for the lower 95% CI of the stability slope and assay variability) [24]. Finally, linear regression modeling was performed on the same stability data sets to estimate the longer-term stability profiles and shelf-life as summarized below.

**Figure 8:**
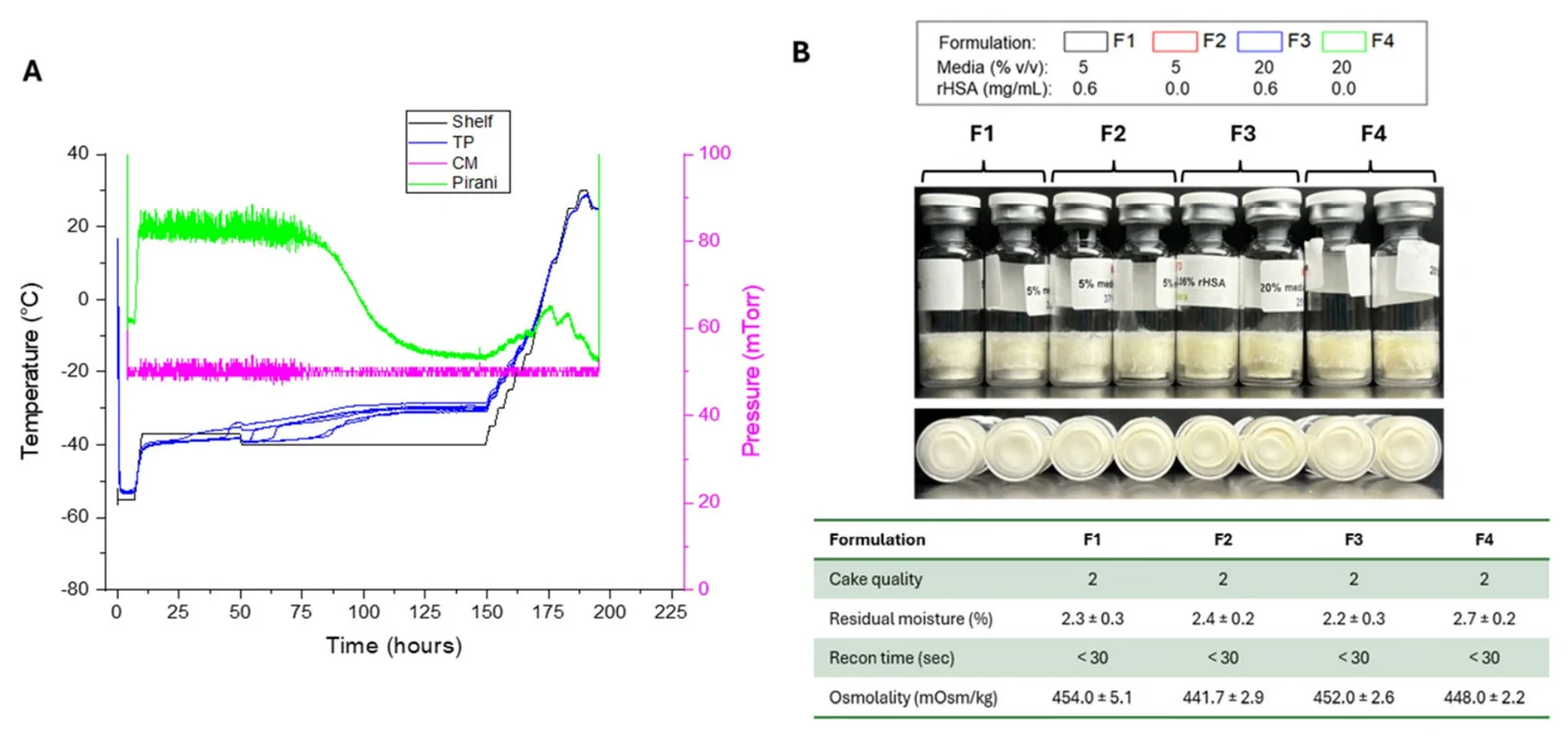
Lyophilization of 5-dose 2X concentrated candidate MR formulation filled into 4R vials and lyophilized in the Lyo Star 4 freeze-dryer as part of the “pilot scale stability study”. The 2X-concentrated candidate MR formulations F1–F4 varied in (1) the volume percent of viral bulk/media (5 vs. 20% *v*/*v*), and (2) the presence vs. absence of rHSA (0.6 mg/mL) in the stabilizer solution (95 vs. 80% *v*/*v*), as indicated in the figure. (**A**) Temperature/pressure as a function of time for the conservative lyophilization cycle used to freeze-dry the samples. (**B**) Cake quality, reconstitution time, and residual moisture content of the lyophilized formulations. The cake quality was assessed by visual observation using a scale of 3 = excellent, 2 = good-to-fair, and 1 = poor. See Table 2 for the estimated composition of the candidate MR formulation.

For this “pilot-scale stability study”, the four variations of the 5-dose, 2X concentrated, candidate MR formulation (F1–F4) were prepared at 4.6 log TCID_50_/mL with a 1.5 mL fill volume (reconstituted to 3 mL WFI resulting in final viral titers at 4.3 log TCID_50_/mL at a 1X candidate MR formulation concentration), filled into 4R vials, and lyophilized in the LyoStar 4 unit using the conservative lyophilization cycle (Figure 8A). The four different variations of the candidate formulation (F1–F4, Figure 8B) were evaluated post-lyophilization for cake quality (good-to-fair), residual moisture (2.2–2.7%), recon times (<30 s), and osmolality values in the range of 441 to 454 mOsm/kg (i.e., the formulation with 0.6 mg/mL rHSA and 20% *v*/*v* media had a higher osmolality value).

**Figure 9:**
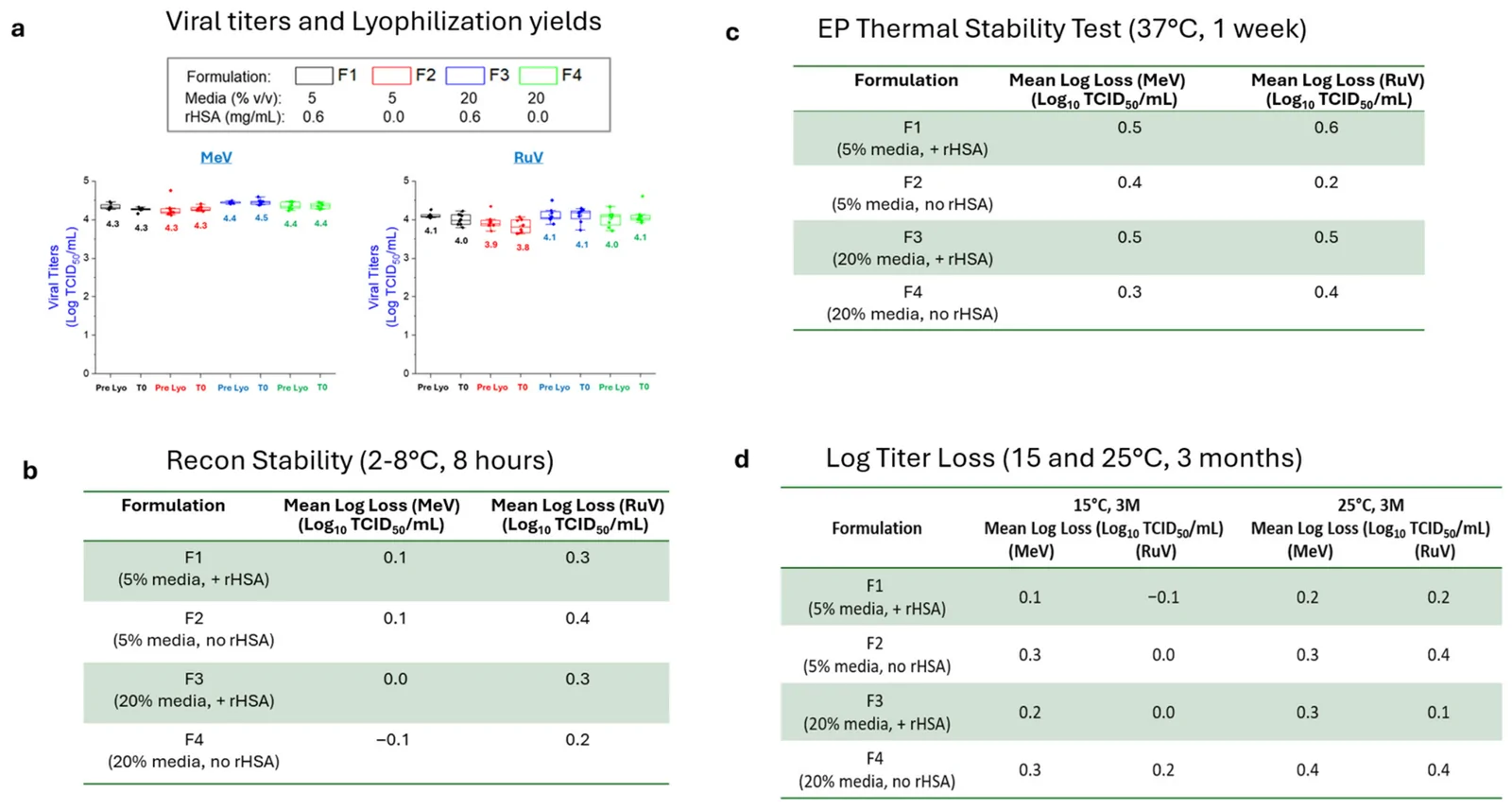
MeV and RuV infectivity titers, lyophilization yields, and accelerated stability profiles in four variations (F1–F4) of the 5-dose, 2X concentrated, lyophilized candidate MR formulation from the “pilot-scale stability study”. Samples were prepared as described in the Figure 8 legend and were assayed for viral titers by PqPCR. (**a**) MeV and RuV viral titers (mean log TCID50/mL) as measured before and after lyophilization. Titer data are presented as box-and-whisker plots (*n* = 8 from 2 vials, each vial analyzed at *n* = 4). (**b**) Reconstitution stability of MeV and RuV (viral titer losses in log TCID50/mL) after 2–8 °C incubation for 8 h vs. freshly reconstituted sample presented as mean values (3–5 vials, each vial analyzed at *n* = 4 with SD range of 0.1 to 0.4). (**c**) EP thermostability results for MeV and RuV (incubation at 37 °C for 1 week) vs. unstressed control (viral titer losses in log TCID50/mL) presented as mean values (2 vials, each vial analyzed at *n* = 4 with SD range of 0.1 to 0.5). (**d**) Accelerated storage stability profiles as MeV and RuV after incubation at 15 and 25 °C for 3 months vs. −80 °C unstressed control, presented as mean values (2–3 vials, each vial analyzed at *n* = 4; SD range of 0.1 to 0.4). See Table 2 for the estimated composition of the candidate MR formulation (the F1–F4 variations included different volume percents of viral bulk and stabilizer solution (the latter either with or without rHSA), as indicated).

The F1–F4 variations of the candidate MR formulation were assayed by PqPCR for viral titers, lyophilization yields (Figure 9a), and the following stability profiles: post-reconstitution (2–8 °C for 8 h; Figure 9b) and accelerated temperature storage (15, 25, and 37 °C; Figure 9c,d). Zero to minimal lyophilization losses of both MeV and RuV were observed in F1–F4 formulations, and all passed the EP thermostability testing criteria (i.e., demonstrated not more than 1.0 log loss after incubation at 37 °C for 7 days [53,54]). In summary, no notable differences were observed across all four variations of the candidate MR formulation (F1–F4) in terms of each of these stability assessments, demonstrating that the following did not have a measurable impact on the candidate MR formulation: (1) the presence vs. absence of rHSA in the stabilizer solution, and (2) the percent volume of cell culture media (5 vs. 20%).

The VVM designation of the candidate MR formulation was estimated based on 3 months stability data at 25 °C (Figure 9d). For a VVM 14 stability rating, the MeV and RuV viral titers should remain above minimum potency for up to 90 days at 25 °C [56]. The stability data demonstrated viral titer losses under these conditions for MeV and RuV, showing reductions of 0.2–0.4 log TCID_50_/mL and 0.1–0.4 log TCID_50_/mL, respectively. These values remained well above the estimated minimum potency specification at expiry (3.3 log TCID_50_/mL, equivalent to 3.0 log TCID_50_ per 0.5 mL dose). Predictions of VVM designations from the available stability data sets at 37 °C were insufficient to support reliable prediction of VVM classification thresholds and were potentially influenced by the biphasic nature of degradation (i.e., non-Arrhenius behavior at high temperature values [24].

**Figure 10:**
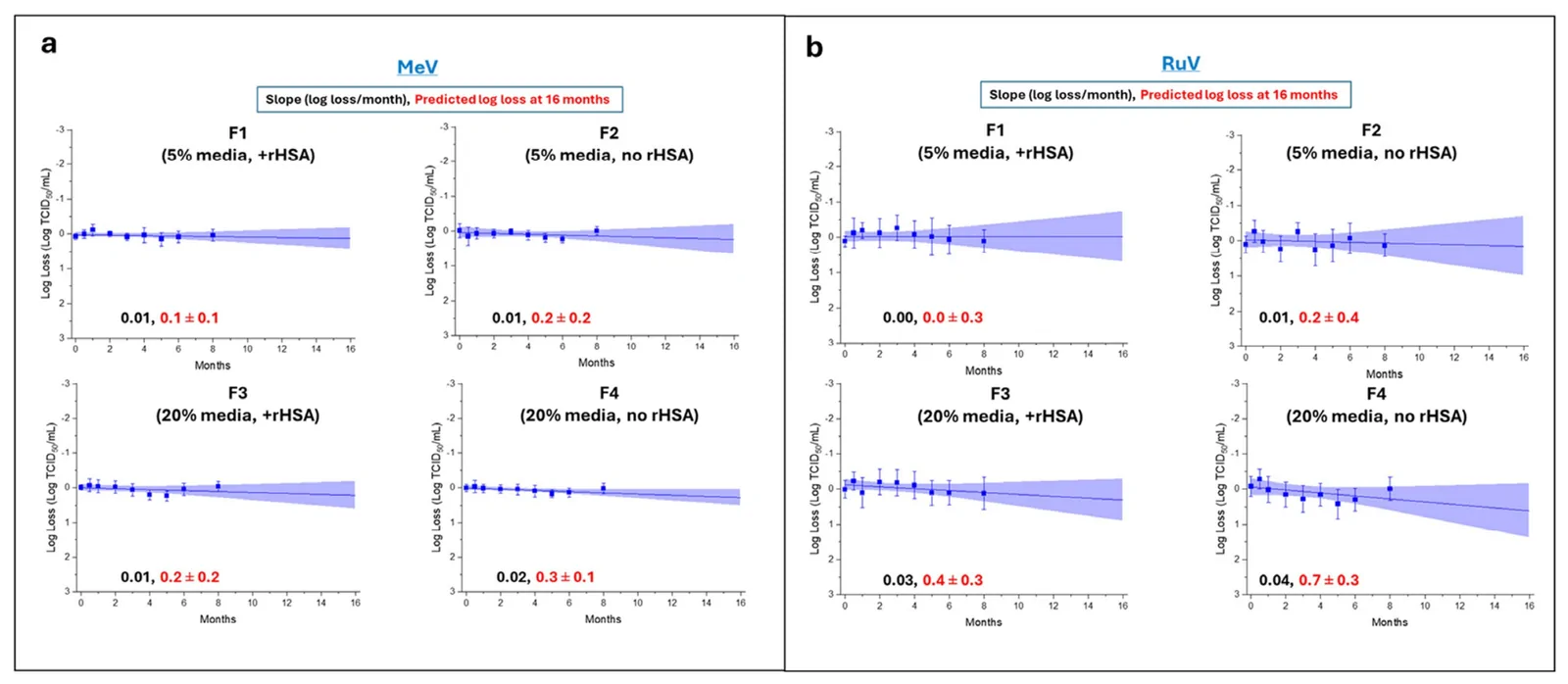
Extrapolation of the real-time 2–8 °C stability data for MeV and RuV in four variations (F1–F4) of the 5-dose, 2X concentrated, lyophilized candidate MR formulation from the “pilot-scale stability study”. Samples were prepared as described in the Figure 8 legend and were assayed for viral titers by PqPCR. Linear regression analysis of the viral titer log loss data for both (**a**) MeV and (**b**) RuV was performed by PqPCR against an unstressed −80 °C control of each formulation. The slope represents log loss per month, with colored bands indicating the 95% confidence interval. Stability data measured up to 8 months were extrapolated two-fold to predict 16-month stability at 2–8 °C. Log loss data are presented as the mean ± 1SD (2 vials, each vial analyzed at *n* = 4). See Table 2 for the estimated composition of the candidate MR formulation (the F1–F4 variations included different volume percents of viral bulk and stabilizer solution (the latter either with or without rHSA) as indicated).

Real-time (2–8 °C) storage stability studies with the same four variations of the MR candidate formulation (F1–F4) were performed up to 8 months. For MeV, no effect on viral stability was noted due to differences in rHSA levels or culture media volume percentages, with comparable regression slopes for all four variations of the formulation in the range of 0.01–0.02 log loss/month (Figure 10a). The estimated mean titer loss values at 8 months for F1–F4 formulations were calculated from the stability slopes to be negligible (~0.1 TCID_50_/mL). The 95% confidence intervals (95% CI) for the samples, accounting for the overall variability of the measured MeV viral titer losses, averaged 0.2 log TCID_50_/mL. A two-fold extrapolation of real-time stability data was performed as described previously for modeling long-term stability of various liquid formulations of a live, attenuated RV3-BB rotavirus vaccine candidate [75]. For MeV, the F1–F4 formulations displayed a predicted mean log loss at 16 months at 2–8 °C in the range of 0.1 to 0.3 TCID_50_/mL, exhibiting no differences between the stability of the F1–F4 formulations given the 95% CI of the stability slopes (Figure 10a).

Similarly, for the RuV component, no differences were observed in the stability profiles in the F1–F4 variations of the candidate MR formulation, with regression slopes in the range of 0.00 to 0.04 mean log (titer) loss/month (Figure 10b). The estimated titer losses at 8 months for the F1–F4 formulations were calculated from the stability slopes to range from 0.0 to 0.3 TCID_50_/mL, with an average 95% CI values of 0.3 log TCID_50_/mL. In summary, excellent 2–8 °C storage stability profiles were observed over 8 months for both the MeV and RuV components in the candidate MR formulation in the 5-dose format lyophilized in a pilot-scale freeze-dryer, and no differences were noted between the four variations (F1–F4) given the experimental error ranges of the stability profiles. For the extrapolation of the stability data for the RuV component, the F1–F4 formulations displayed a predicted mean log loss at 16 months at 2–8 °C in the range of 0.0 to 0.7 TCID_50_/mL (Figure 10b). The predicted mean RuV stability slopes in the F3 and F4 formulations (20% media) showed a trend toward higher titer losses (0.03-0.04 log loss/month) vs. the F1 and F2 (5% media) formulations (0.00–0.01 log loss/month). These differences are likely minor given the overall variability of the extrapolation methodology, and additional real-time stability data were needed to assess these trends.

**Figure 11:**
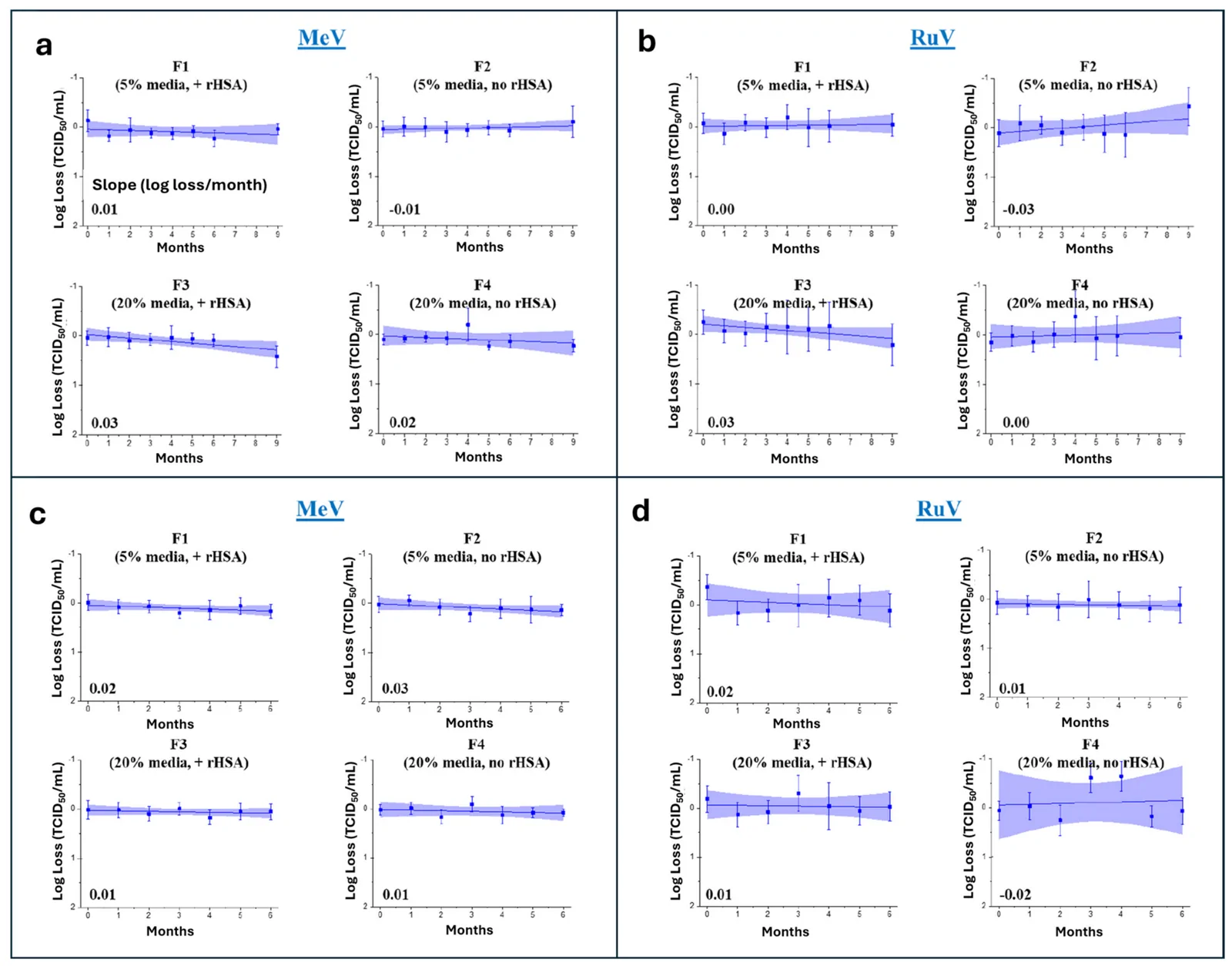
Real-time (2–8 °C) stability studies with four variations (F1–F4) of the single-dose, 2X concentrated candidate MR formulation lyophilized in 4R vials in a lab-scale freeze-dryer. The F1–F4 variations of the single-dose, 2X-concentrated candidate MR formulation were filled into 4R vials, lyophilized in the benchtop VirTis unit, incubated under different conditions, and assayed for viral titers by PqPCR. Linear regression analysis of log loss of viral infectivity titers vs. an unstressed −80 °C control is presented in panels (**a**) MeV and (**b**) RuV up to 9 months at a target viral titer (4.3 log TCID_50_/mL post-reconstitution), and (**c**) MeV and (**d**) RuV up to 6 months at a higher target viral titer (4.8 log TCID_50_/mL post-reconstitution). The slope represents log loss per month, with colored bands indicating the 95% confidence interval. Log loss data are presented as the mean ± 1SD (2 vials, each vial analyzed at *n* = 4). See Table 2 for the estimated composition of the candidate MR formulation (the F1–F4 variations included different volume percents of viral bulk and stabilizer solution (the latter either with or without rHSA) as indicated).

To further evaluate these MR stability trends described above, two additional lab-based storage stability studies were performed using single-dose (0.65 mL), 2X-concentrated candidate MR formulation lyophilized in the benchtop VirTis freezer-dryer. The first study was set up with the same four variations of the formulation (F1–F4) at a lower target viral titer (4.3 log TCID_50_/mL post-reconstitution). The second stability study was similar to the first study except at a higher target viral titer (4.8 log TCID_50_/mL post-reconstitution). The F1–F4 variations of the candidate MR formulation were stored at 2–8 °C for up to 9 and 6 months, respectively, and compared with unstressed −80 °C controls using linear regression analysis of log loss of viral infectivity titers as measured by PqPCR assay. Results showed none to minimal loss in both MeV (Figure 11a,c) and RuV (Figure 11b,d) titers over the study period. The stability trends across the samples were not notably affected by the presence or absence of rHSA or by differences in cell culture media composition, as indicated by the overlapping 95% confidence intervals of the stability slopes. In summary, these two additional lab-scale stability studies showed no notable differences in variations of the candidate MR formulation during real-time (2–8 °C) stability profiles of both MeV and RuV components.

## 4. Discussion and Future Work

The overall goals of the experimental studies described in this case study were to design, evaluate, and “de-risk” the development of a stable, lyophilized, multidose formulation for an MR vaccine candidate targeted for local production in Africa and for use in LMICs. The lab-scale MR vaccine formulation/lyophilization development work described herein was performed using concentrated MeV and RuV bulks produced by our collaborators in MRC-5 cells by a new manufacturing process (see Introduction [16,17]). The bulk manufacturing process used the HIP-Vax^®^ platform from Batavia Biosciences to produce high-titer MeV and RuV bulks. Virus-producing cells were grown on a fixed-bed matrix inside a scale-X™ bioreactor, integrated within the Univercells NevoLine™ Upstream contained microfacility. A continuous perfusion process with tangential flow filtration was used to collect and concentrate the viral harvest, resulting in higher yields than conventional production methods. Specific formulation goals were guided by criteria set out in the target product profile (TPP), as developed by a cross-functional team with our collaborators at BBS (The Netherlands) and IPD (Senegal, West Africa), and as supported by grants from the Gates Foundation (see Introduction [18,20]).

As a starting point for the multidose MR vaccine formulation development, we evaluated the composition of an off-patent formulation of a commercial, single-dose trivalent MMR vaccine using publicly available information. Modifications and alterations were then made to optimize the composition based on differences in presentation (multidose), viral bulk manufacturing processes (more concentrated viral bulks produced in different cells and culture media), and considerations to lower costs (removing or minimizing expensive additives). In summary, a candidate MR formulation composition was defined, along with adaptations to accommodate a multidose (5-dose) format including a 2X concentrated version for lyophilization (reconstituted to a 1X concentration for administration) and using a larger, 4R glass vial for primary packaging. Next, suitable lyophilization cycles were developed using both, with lab-scale and pilot-scale freeze-dryers, and real-time and accelerated stability studies were conducted to estimate shelf-life and VVM designation.

To support future technology transfer and manufacturing of this lyophilized, multidose MR vaccine candidate, as well as to provide general guidance for future live-virus vaccine (LVV) development work for candidates targeted for use in LMICs, the following key conclusions and lessons-learned encountered during this case study have been organized below into the following four technical topics: (1) the key role of viral infectivity assays to assess stability profiles, (2) challenges determining the “stability window” to estimate the required release dose to ensure minimum potency at expiry, (3) inter-relationships between viral bulk composition, stabilizer composition, and lyophilized drug product stability, and (4) challenges during freeze-drying of larger fill-volumes of a multidose formulation with poor freeze-drying properties.

### 4.1. Key Role of Viral Infectivity Assays During MR Formulation Development

In this work, the PqPCR assay was used to measure MeV and RuV infectivity titers during both formulation development and storage stability studies. PqPCR is a single-assay setup that measures the infectivity titers of both MeV and RuV in a 96-well plate format using a single cell line (Vero cells). In contrast, the quality control (QC) TCID_50_ assay is far more time-consuming, labor-intensive, and requires two separate assay setups (one each for RuV and MeV, the latter requiring preparation, qualification, and use of anti-Rubella antisera).

We have previously established that MeV and RuV stability trends correlate well between PqPCR and TCID_50_ assays [40]. The PqPCR method was thus chosen for this MR vaccine formulation development work due to its higher-throughput, shorter virus incubation times, less resource-intensive nature, and ability to accommodate substantially more replicates per sample (thus improving the precision of potency measurements as well as more formulation samples per experiment). Although PqPCR enabled more rapid MR multidose formulation development, there are two drawbacks we have identified: (1) it is a more expensive assay to run in terms of reagents and equipment, and (2) it is not the official QC assay for determining the viral infectivity titers of commercial MR-containing vaccines.

For the latter, there remains a question whether the results from this lab-scale work will be confirmed during subsequent technology transfer using the TCID_50_ assay in a QC environment. Although we have observed previously that the PqPCR and TCID_50_ assays correlated well in terms of stability profiles (i.e., percent loss of titer during storage relative to time zero results [40]), we also noted that the absolute titer values (log TCID_50_ /mL) may differ between the two assays for one or both viruses (e.g., MeV, RuV). Such differences could be addressed, and potentially eliminated or accounted for, during future assay validation work since there are examples, with other live virus vaccines, of validating PqPCR as a QC test for viral infectivity titers and thus replacing the TCID_50_ assay in a QC environment [76].

### 4.2. Challenges Associated with Determining the “Stability Window” to Estimate Release Dose Required to Ensure Minimum Potency at Expiry

Real-time and accelerated stability studies were performed with the four variations (F1–F4) of the five-dose candidate lyophilized MR formulation (2X concentrated version filled at 1.5 mL into 4R vials, lyophilized in the pilot-scale freeze-dryer, and reconstituted to 3 mL with water). The viral infectivity titers were determined by PqPCR assay, and the following analyses were performed: (1) rank-order the lyophilization yields and stability profiles of the MeV and RuV components in the four samples at different temperatures, (2) estimate 2–8 °C shelf-life by extrapolation of stability data, (3) estimate the VVM designation by evaluating the accelerated stability data, and (4) estimate the “stability window” between release and expiry doses.

The overall results showed promising lyophilization yields as well as accelerated and real-time stability profiles for the MeV and RuV in the F1–F4 variations of the MR candidate formulation. No differences were noted due to the presence/absence of rHSA in the stabilizer solution, with potentially some differences for the RuV component when varying the media percent volume from 5% to 20% (*v*/*v*). Two additional stability studies, however, using the 2X concentrated, single-dose candidate MR formulations lyophilized in a benchtop VirTis freeze-dryer, were performed and demonstrated RuV stability profiles independent of rHSA or media differences. Using MR stability data during storage for up to 8 months at 2–8 °C, we estimated projected losses at 16 months via modeling and extrapolating of the stability data as described previously [75], for both MeV and RuV components. The 2–8 °C trends are promising, and a longer shelf life of 18–24 months seems possible (to be confirmed with additional stability data). Finally, a VVM-14 designation was estimated using 25 °C stability data up to 3 months, showing that the F1–F4 variations of the MR candidate formulation remained well above the estimated minimal expiry dose. Based on these data, the MR formulation with the 5% (*v*/*v*) bulk and 95% (*v*/*v*) stabilizer solution without rHSA (i.e., the F2 formulation) was selected as the most promising candidate to lower costs (see Section 4.3).

To determine the “stability window”, we started with the assumption that to ensure vaccine efficacy, at least 1000 TCID_50_ /dose of both MeV and RuV are required at expiry (including reconstitution stability after storage as a lyophilized dosage form [52]). Based on regulatory expectations, this minimum titer value is not the mean value, but rather the lower 95% CI of the stability slope, a value determined by assay variability [24,77]. Finally, the release dose can be estimated based on not only storage stability results after long-term storage at 2–8 °C, but also manufacturing capabilities (i.e., highest titer bulks that can be produced at large scale), analytical capabilities (i.e., variability of the validated viral infectivity assays run in the QC labs), and clinical experience of safety limits (highest dose that can be administered) as described previously [77]. Based on the fill titers used in this work, the “stability window” estimate required less than one log loss of viral infectivity titers during storage (i.e., to define the shelf-life at 2–8 °C where the expiry dose would be reached). For the two viral components of the MR candidate multidose formulation, both displayed excellent storage stability up to 8 months and are anticipated to have an 18–24 month shelf-life at 2–8 °C.

At the same time, the results in this work were obtained with single lots of concentrated MeV and RuV bulks (produced at BBS) and measured by PqPCR run in an R&D lab. Thus, the final “stability window” estimation for the MR lyophilized multidose formulation candidate must be confirmed (and potentially adjusted) in the future based on (1) results from multiple lots of concentrated MeV and RuV bulks produced at IPD, and (2) assay variability estimates of the two TCID_50_ assays run at IPD.

### 4.3. Inter-Relationships Between Viral Bulk Composition, Stabilizer Solution Composition, and Stability of the Lyophilized MR Drug Product

The inter-relationships between the (1) composition of the concentrated MeV and RuV bulks, (2) the composition of the candidate MR formulation, and (3) the stability of the lyophilized multidose MR drug product were evaluated in this work. The 5-dose candidate MR formulation composition (amount of each additive per dose) was estimated by assuming 95% (*v*/*v*) stabilizer solution and 5% (*v*/*v*) virus bulks/culture media. In addition, a multidose formulation and lyophilization process was defined using a 2X concentrated version of this formulation, a 1.5 mL fill volume into 4R vials, lyophilization, and then reconstitution with 3 mL WFI (to a final 1X formulation composition and the target viral titers).

The starting point for the candidate MR multidose formulation composition was based on the off-patent, publicly available composition of the commercial, single-dose, lyophilized M-M-R^®^II vaccine. This baseline composition was then adapted as a multidose presentation using concentrated MeV and RuV viral bulks (produced by a manufacturing process using different host cells and in different culture media). We identified and evaluated two components of the candidate MR multidose lyophilized formulation composition that provided the opportunity to lower costs: First, concentrated MeV and RuV bulks allowed for 5% (*v*/*v*) viral bulk/media in the MR formulation, which improved the lyophilization properties. Second, the 95% (*v*/*v*) stabilizer solution did not require the addition of rHSA, because the rHSA in the stabilizer solution (up to the levels reported in the M-M-R^®^II vaccine of 300 mcg/dose) did not affect the MeV or RuV stability profiles. Since the concentrated MeV bulks contained no rHSA while the RuV bulks had minimal carryover of rHSA (high titer bulks result in larger dilution in the drug product, we estimated only very low levels of rHSA in the candidate MR formulation (approx. 1–10 mcg/dose). In summary, significant cost savings were achieved by reducing the volume percent of viral bulks/culture media and by not adding rHSA to the stabilizer solution (combined with minimal levels carried over from the concentrated viral bulks).

### 4.4. Lyophilization Challenges with Larger Fill-Volumes of a Multidose MR Formulation with Poor Freeze-Drying Properties

In this work, we encountered numerous lyophilization process challenges with the larger fill volume required for a multidose presentation of the candidate MR formulation, especially because of the poor freeze-drying properties of the viral bulks and some of the stabilizing excipients. The 5-dose presentation (0.5 mL/dose) requires an overfill resulting in a final volume of 3 mL post-reconstitution (to ensure 5 doses can be extracted from the vial). Moreover, the composition of the candidate MR formulation contained several additional components that are challenging to freeze-dry, including (1) culture media/viral bulks (a complex mixture of salts, amino acids, and other additives) and (2) low glass transition temperature values (*T_g_*′) of stabilizers such as sorbitol and phosphate buffer. Based on these considerations, hydrolyzed gelatin is also a key additive to enable successful freeze-drying of this formulation, as it not only provides bulking properties to facilitate cake integrity during lyophilization, but it is also a well-known stabilizer of MeV and RuV in combination with sorbitol [78,79].

The *T_g_*′ value of the candidate MR formulation is −52 °C, which requires a very low shelf temperature for freezing as well as primary drying (to remove bulk water), which in turn results in very slow sublimation rates and long lyophilization cycles. These long and cold freeze-drying cycles are referred to in this work as “conservative lyo cycles”. Moreover, customization of these conservative lyophilization cycles was required for the candidate MR formulation in different vial sizes, fill volumes, or when using different freeze-drying units. Specifically, modified conservative lyo cycles were developed for a single-dose presentation in 2R vials (i.e., lab-scale vs. LyoStar 2 pilot-scale units), 5-dose presentation in 4R vials (i.e., LyoStar 2 vs. 4 pilot units), and a 5-dose presentation in 6R vials (LyoStar 2). Next, we evaluated the use of a 2X concentrated version of the candidate MR formulation (for both excipients and the target viral titers). This presentation was filled at 1.5 mL, lyophilized, then reconstituted with 3 mL WFI (resulting in 1X formulation concentration at the target viral titer values for MeV and RuV). Based on these considerations, a 5-dose candidate MR formulation (filled at 2X concentration at 1.5 mL), filled into 4R vials, and lyophilized in the LyoStar 4 was selected as the preferred conservative lyophilization cycle (total cycle time of ~8 days).

Two initial tech transfer lyo cycles for this candidate MR multidose formulation (total cycle time reduced to 7 and 6 days in the LyoStar 4 unit, respectively) were evaluated by optimizing the freeze-drying parameters (i.e., freezing rates, and temperature/pressure/time settings for primary and secondary drying steps). Initial proof-of-concept was established, including cake quality, residual moisture, reconstitution time, and viral titer loss after lyophilization and thermal stress (7 days at 37 °C). Although further optimization is certainly possible, it may be challenging to further shorten the freeze-drying cycle times given the very low glass transition temperature (*T_g_*′ = −52 °C) of the candidate MR formulation. Finally, the translation of the optimized tech transfer lyophilization cycle in the LyoStar 4 unit in a laboratory setting will need to be further evaluated in a larger, manufacturing-scale freeze-drying unit. This assessment will need to be part of a future drug product technology transfer project for the scale-up and manufacturing of this multidose lyophilized MR vaccine formulation at IPD, Senegal.

## 5. Conclusions

This case study describes lab-scale formulation development of a stable, lower-cost, five-dose lyophilized bivalent MR vaccine candidate targeted for future technology transfer and local manufacturing in Africa. As a starting point, this work used (i) higher-titer, concentrated MeV and RuV bulks produced in MRC-5 cells by a new manufacturing process, (ii) an off-patent, publicly-available formulation composition of a single-dose trivalent MMR vaccine, and (iii) a high-throughput RT-qPCR assay to measure MeV and RuV infectivity titers. The key characteristics of the lyophilized multidose MR formulation included (i) use of a reduced viral bulk/media volume of 5% (*v*/*v*), (ii) removal of rHSA from the 95% (*v*/*v*) stabilizer solution, and (iii) use of a five-dose formulation filled into 4R glass vials, lyophilized at 2X concentration (1.5 mL fill) and reconstituted with 3 mL of diluent to a 1X concentration. The down-selected candidate multidose MR formulation showed minimal losses after lyophilization, during real-time stability conditions, and after reconstitution (2–8 °C for 8 h). For example, little or no titer losses were observed over 8 months at 2–8 °C, which supports a projected shelf-life of at least 16 months. In addition, based on limited 25 °C stability data, an estimated VVM14 designation was determined. Finally, the formulation also met the European Pharmacopoeia thermostability criterion (≤1 log reduction of viral titers after 7 days at 37 °C). Future technology transfer should focus on confirming the results with the QC viral infectivity assay (TCID_50_) and multiple bulk lots produced at large-scale as well as further shortening the freeze-drying cycle time and scaling-up to a manufacturing-scale lyophilizer.

## Figures and Tables

**Table 1:** Summary of Target Product Profile (TPP) parameters related to vaccine dosage form and stability requirements to guide formulation development of a multidose, lyophilized bivalent MR vaccine candidate targeted for technology transfer and local production at Institut Pasteur, Dakar (IPD) in Senegal, Africa.

Attributes		Description	References
Vaccine Dosage Form Requirements	Dosage form	Lyophilized vaccine in a stoppered, crimped glass vial which will be packaged with a diluent (for vaccine reconstitution) in a separate stoppered, crimped glass vial	[50,51]
	Number of vaccine doses per vial	Five	[50,51]
	Route	IM or SC to match prequalified WHO vaccines for MR	[50,51,52]
	Expiry dose	3.0 log TCID_50_/dose as described for live MeV and RuV vaccines	[30]
Stability Requirements	Storage temperature and shelf-life	Maintain minimum expiry dose after storage up to 2 years at the recommended storage temperature of 2–8 °C	[52]
	Accelerated stability profiles	≤1 log loss (37 °C, 7 days) as defined per EP pharmacopeia for live Measles, Mumps, and Rubella vaccine (live)	[53,54,55]
	Reconstitution stability	Maintain minimum expiry dose after reconstitution with diluent followed by storage for 8 h at 2–8 °C	[52]
	Vaccine Vial Monitor (VVM)	VVM designation of Type 14 as defined per WHO technical document	[50,51,56]

## Data Availability

The datasets generated and/or analyzed in this work, as well as Supplementary Materials, are available in the KU ScholarWorks repository: https://doi.org/10.17161/1808.39604 [80].

## Supplementary Materials

The following supporting information can be downloaded at: https://www.mdpi.com/article/10.3390/vaccines14100900/s1, Supplementary Table S1: Conservative lyophilization cycle parameters for the pilot-scale freeze-drying the 5-dose, 2X concentrated, lyophilized candidate MR formulation; Supplementary Table S2: Initial optimization of lyophilization cycle parameters for the pilot-scale LyoStar4 unit for freeze-drying the 5-dose, 2X concentrated, lyophilized candidate MR formulation.

## Abbreviations

AFRO	The WHO African Region
BBS	Batavia Biosciences
Bio-E	Biological E. Limited
CMC	Chemistry, Manufacturing, and Controls
CRS	Congenital Rubella syndrome
FBS	Fetal bovine serum
GMP	Good manufacturing practices
IA2030	Immunization Agenda 2030
IPD	Institut Pasteur de Dakar
KU	The University of Kansas
LMICs	low- and middle- income countries
LVVs	Live viral vaccines
mDSC	Modulated Differential Scanning Calorimetry
MEM	Minimum essential medium
MEV	Measles
MMR	Measles, Mumps, and Rubella
MR	Measles and Rubella
MRC-5	Medical Research Council cell strain 5 (human diploid fibroblasts)
MMRV	Measles, Mumps, Rubella, and Varicella
PqPCR	Potency quantitative polymerase chain reaction
RT-qPCR	Reverse transcription quantitative polymerase chain reaction
RuV	Rubella
SIIPL	Serum Institute of India Private Limited
SSC	System suitability controls
TCID50	50% Tissue Culture Infectious Dose
*T_g_*	Glass transition temperature of the lyophilized drug product
*T_g_*′	Glass transition temperature of the maximally freeze-concentrated formulated liquid
TPP	Target Product Profile
US FDA	United States Food and Drug Administration
VAFC	Vaccine Analytics and Formulation Center
VVM	Vaccine vial monitor
WFI	Water for injection
WHO	World Health Organization
WI-38	Wistar Institute-38 human diploid fibroblast cell strain
