# Supplementary material for: Case Study on Formulating a Multidose Lyophilized Measles-Rubella (MR) Vaccine Candidate for Local Manufacturing in Africa: Lab-Scale Results and Lessons Learned for Future Technology Transfer
Source: Vaccines (Basel). 2026 Oct 14;14(10):900. doi: 10.3390/vaccines14100900 (PMC13709750; doi:10.3390/vaccines14100900)
Supplement: Supplementary file 1 [file vaccines-14-00900-s001.zip › vaccines-4572114-supplementary.pdf]

Case Study on Formulating a Multidose Lyophilized Measles-Rubella (MR) Vaccine Candidate for Local Manufacturing in Africa: Lab-Scale Results and Lessons-Learned for Future Technology Transfer

Kathryn Secrist[1,@,#,], Prashant Kumar[1,@], Michael Z. Wang[1], Soraia Saleh-Birdjandi[1], Sagar Salave[1], Ozan S. Kumru[1], Ngana Salem Mberkadji[2], Fama Sakho[2], Sangeeta B. Joshi[1,*], David B. Volkin[1,*]

[1] Department of Pharmaceutical Chemistry, Vaccine Analytics and Formulation Center, University of Kansas, Lawrence, Kansas 66047, USA

[2] Vaccine Research Center (VRC) at Institut Pasteur de Dakar (IPD), 36 Avenue Pasteur, B.P. 220, Dakar, Senegal

***Corresponding authors:** Multidisciplinary Research Building, 2030 Becker Dr., Lawrence, KS 66047, USA. (SBJ) Phone: 785-864-3356; Email: joshi@ku.edu; (DBV) Phone: 785-864-6262; Email: volkin@ku.edu

[@] These authors contributed equally

[#] Current address: Boehringer Ingelheim Animal Health, St Joseph, MO 64506, USA

Keywords: Measles, Rubella, Vaccine, Formulation, Stability, Lyophilization, qPCR, Viral Infectivity Assay, Excipients

Supplementary materials

a

	Freeze		Primary drying			Secondary drying									Final
**Step**	1	2	3	4	5	6	7	8	9	10	11	12	13	14	15
**Shelf Temp (°C)**	-55	-55	-55	-37	-40	-35	-30	-25	-20	-15	10	25	30	25	4
**Ramp Rate (°C/min)**	--	--	--	0.1	0.1	0.05	0.05	0.05	0.05	0.05	0.05	0.05	0.05	0.05	1.0
**Time (hr)**	--	2	3	30	109	2	2	2	2	2	2	3	3	3	Hold
**Vacuum (mT)**	--	--	32	32	32	32	32	32	32	32	32	32	32	32	32

b

	Freeze		Primary drying			Secondary drying									Final
**Step**	1	2	3	4	5	6	7	8	9	10	11	12	13	14	15
**Shelf Temp (°C)**	-55	-55	-55	-37	-40	-35	-30	-25	-20	-15	10	25	30	25	4
**Ramp Rate (°C/min)**	--	--	--	0.1	0.1	0.05	0.05	0.05	0.05	0.05	0.05	0.05	0.05	0.05	1.0
**Time (hr)**	--	3	3	30	109	2	2	2	2	2	2	3	3	3	Hold
**Vacuum (mT)**	--	--	50	50	50	50	50	50	50	50	50	50	50	50	32
**PVG/CM Diff**	--	--	--	--	10	--	--	--	--	--	--	--	--	--	--

Supplementary Table S1. Conservative lyophilization cycle parameters for the pilot-scale freeze-drying the 5-dose, 2X concentrated, lyophilized candidate MR formulation. Samples were filled into 4R vials (1.5 mL), lyophilized and then reconstituted using 3.0 mL WFI). (A) cycle parameters for the LyoStar 2 unit, and (B) LyoStar 4 with both cycles having a total time ~8 days.

a

	Freezing		Primary drying			Secondary drying									Final
**Step**	1	2	3	4	5	5	6	7	8	9	10	11	12	13	14
**Shelf Temp (°C)**	-55	-55	-55	-37	-40	-35	-30	-25	-20	-15	10	25	30	25	4
**Ramp Rate (°C/min)**	--	--	--	0.1	0.1	0.05	0.05	0.05	0.05	0.05	0.05	0.05	0.05	0.05	1
**Time (hours)**	--	3	3	30	84	2	2	2	2	2	2	3	3	3	Hold
**Vacuum (mT)**	--	--	50	50	50	50	50	50	50	50	50	50	50	50	50
**PVG/CM Diff**	--	--	--	--	5	--	--	--	--	--	--	--	--	10	

b

	Freezing	Primary drying		Secondary drying								
**Step**	1	2	3	5	6	7	8	9	10	11	12	13
**Shelf Temp (°C)**	-55	-55	-37	-35	-30	-25	-20	-15	10	25	30	25
**Ramp Rate (°C/min)**	45	0	180	100	100	100	100	100	500	300	300	100
**Hold Time (hr)**	3	3	40	2	2	2	2	2	2	3	3	3
**Vacuum (mT)**	--	50	50	50	50	50	50	50	50	50	50	50
**PVG/CM Diff**	--	--	10	--	--	--	--	--	--	--	--	--

Supplementary Table S2. Initial optimization of lyophilization cycle parameters for the pilot-scale LyoStar4 unit for freeze-drying the 5-dose, 2X concentrated, lyophilized candidate MR formulation. Samples were filled into 4R vials (1.5 mL), lyophilized and then reconstituted using 3.0 mL WFI). (A) first tech-transfer lyophilization cycle (total time ~7 days) and, (B) second tech-transfer lyophilization cycle (total time ~6 days).
